# The CRF‑CRFR1 Axis Mediates Prenatal Fear Stress‑Induced Hippocampal Mitochondrial Damage in Offspring via MAM Remodeling and Calcium Overload

**DOI:** 10.1007/s10571-026-01780-0

**Published:** 2026-07-19

**Authors:** Yu-jie Li, Li-ping Yang, Yun-yu Wang, Tian-ning Gu, Yi-chi Zhang, Lan Zhang, Shan Cao, Jun-lin Hou

**Affiliations:** 1 Department of Biochemistry, School of Medicine, Henan University of Chinese Medicine, Zhengzhou, 450046 P.R. China; 2 Department of Integrated Traditional Chinese and Western Medicine, School of Traditional Medicine, Henan University of Chinese Medicine, Zhengzhou, 450046 P.R. China; 3 Graduate School, Henan University of Chinese Medicine, Zhengzhou, 450046 P.R. China; 4 The First School of Clinical Medicine, Henan University, Kaifeng, 475004 P.R. China; 5 School of Medicine, Henan University of Chinese Medicine, Zhengzhou, 450046 P.R. China

**Keywords:** Prenatal fear stress, Hippocampal mitochondrial damage, CRFR1, PLCβ1, Mitochondria‑associated ER membrane

## Abstract

Mitochondrial dysfunction has been recognized as one of the three hallmark biological features of autism spectrum disorder. This study is intended to investigate the molecular mechanisms underlying prenatal psychological fear stress‑induced hippocampal mitochondrial damage in offspring. Bioinformatics analysis revealed that the calcium signaling pathway, particularly the phospholipase C beta 1(PLCβ1)- inositol 1,4,5‑trisphosphate receptor (IP3R)- voltage‑dependent anion channel 1(VDAC1) pathway, may play a key role in prenatal stress‑induced hippocampal mitochondrial damage in offspring. To validate this, we examined pathway activation in the offspring hippocampus of prenatal fear stressed rats and in corticotropin-releasing hormone (CRH; also known as CRF in rodents) overexposed SH‑SY5Y cells, along with mitochondrial calcium levels in the cells. Prenatal fear stress induced depressive-like behavior and HPA axis activation in pregnant dams, and reduced survival and growth in offspring. Placental and neonatal brain CRF levels were elevated. At the early socialization stage (Postnatal day 21–30), model offspring showed normal basal but exaggerated stress-induced HPA responses. Their hippocampus exhibited expanded MAM coverage, reduced ER-mitochondria distance, and upregulated PLCβ1, IP3R1, VDAC1 expression, along with increased GRP75‑VDAC1 co‑localization, confirming MAM remodeling at the molecular level. In vitro, CRH (20 µM for 48 h or 5–20 µM for 96 h) inhibited SH-SY5Y cell proliferation, upregulated CRH receptor 1(CRHR1), PLCβ1, VDAC1, and increased mitochondrial calcium; these effects were reversed by the CRHR1 antagonist CP376395 or PLCβ1 knockdown. Furthermore, the MCU inhibitor DS16570511 partially reversed CRH‑induced mitochondrial calcium elevation and proliferation inhibition, suggesting that mitochondrial calcium overload contributes to the proliferation inhibition. Collectively, these results suggest that prenatal fear stress may, via the CRF-CRFR1 axis, influence the PLCβ1-IP3R-VDAC1 pathway, inducing MAM remodeling and mitochondrial calcium overload, thereby contributing to offspring hippocampal neuronal damage.

**Graphical Abstract:**

**Figure:**
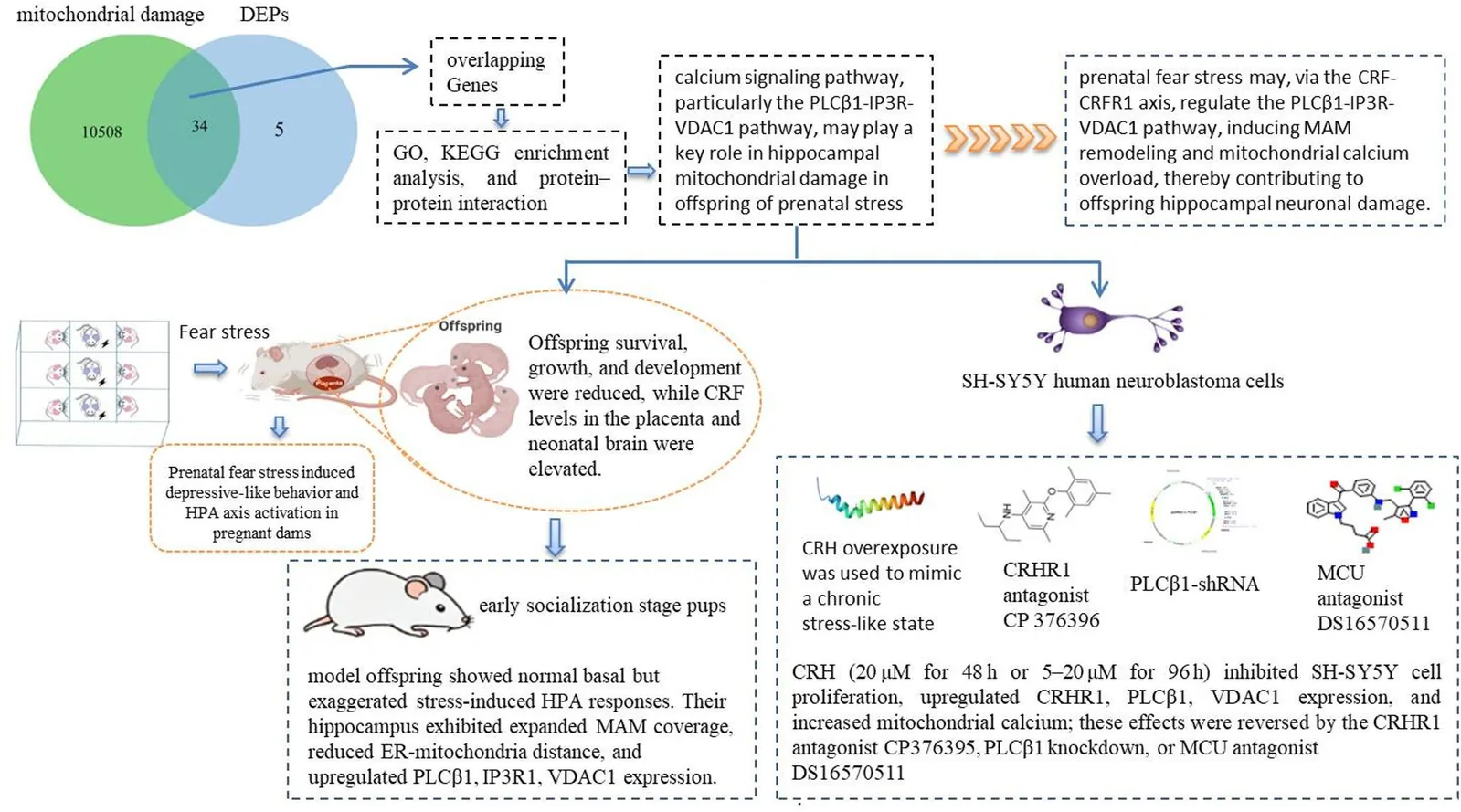

**Supplementary Information:**

The online version contains supplementary material available at https://doi.org/10.1007/s10571-026-01780-0.

## Background

The “developmental origins of health and disease” hypothesis has gradually been recognized by the medical community in recent years. This theory postulates that the embryonic stage is a critical and sensitive developmental period, during which changes occur in the intrauterine environment, such as nutritional deprivation or excessive exposure to glucocorticoids, can exert profound long-term impacts on fetal growth and development. These effects include multiple physiological systems, including the central nervous system (CNS), autonomic nervous system (ANS), neuroendocrine system (especially the hypothalamic pituitary adrenal axis, HPA axis), cardiovascular and immune systems (Van den Bergh et al. 2020). Prenatal psychological stress can induce alterations in neurogenesis during both the embryonic and postnatal developmental stages, thereby increasing the susceptibility of offspring to depression and other mental illnesses in their future lives (Belnoue et al. 2013; Salmina et al. 2021). Mitochondria play a vital role in nearly all eukaryotic cells, serving as the primary site for oxidative phosphorylation and ATP generation. Beyond energy production, they are central to diverse cellular processes, including redox homeostasis, calcium signaling, autophagy regulation, and apoptotic pathway activation (Frye et al. 2021). Mitochondrial dysfunction has been recognized as one of the three hallmark biological features of autism spectrum disorder (ASD). Clinical observations reveal that children with ASD and comorbid neurodegenerative conditions frequently demonstrate abnormal mitochondrial function, characterized by: elevated respiratory rates, increased oxygen consumption, and dysregulated mitochondrial autophagy pathways (Singh et al. 2020; Napoli et al. 2018). Prenatal stress induces offspring mitochondrial dysfunction, correlating with social behavioral deficits (Alhassen et al. 2021). Our previous study also shown that fear stress during pregnancy can lead to mitochondrial damage and abnormalities in oxidative phosphorylation function in the hippocampal of offspring (Li et al. 2020). However, the specific biochemical pathways through which prenatal fear stress brings about mitochondrial impairment in the hippocampus offspring remain unclear.

In the present study, we used “mitochondrial damage” as the keyword to obtain target genes related to mitochondrial damage from GeneCards and NCBI databases. Then, we selected the differentially expressed proteins (DEPs) from the results of the previous hippocampal proteomic analysis (Li et al. 2020). Subsequently, the Wei Sheng Xin website (https://www.bioinformatics.com.cn/) was utilized to construct a Venn diagram for identifying overlapping genes between mitochondrial damage and DEPs in order to conduct an enrichment analysis on the genes related to mitochondrial function in hippocampal proteomics. We validated the findings through western blotting and a sequence of other biochemical analyses. Such results could offer vital insights into the molecular association between the prenatal stress and mitochondrial damage in hippocampus of offspring. Such findings might potentially contribute to the development of preventive and therapeutic strategies for disorders linked to prenatal psychological stress.

## Materials and Methods

### Bioinformatics Analysis

Using “mitochondrial damage” as the keyword, collect targets related to mitochondrial damage using Genecard databases (https://www.genecards.org/, selected only the Relevance score > 1 proteins) and NCBI databases (https://www.ncbi.nlm.nih.gov/). Based on our previously published hippocampal proteomic data (Li et al. 2020), we re‑screened DEPs using a more stringent cutoff (fold change ≥ 1.5 or ≤ 0.67, *p* ≤ 0.05), compared with the 1.2‑fold threshold used in our prior publication. The overlapping genes between the two sets were then identified via Venn diagram. A series of bioinformatics analyses were performed on these overlapping genes: The protein–protein interaction (PPI) network was constructed by the STRING database (Szklarczyk et al. 2021); the refined functional annotation of the overlapping gene set was conducted using the Gene Ontology (GO) database; and the significantly enriched signaling pathways were predicted via the Kyoto Encyclopedia of Genes and Genomes (KEGG) database (Xu et al. 2019; Liu et al. 2019; Bindea et al. 2009).

### Animal Care and Ethics Statement

Thirty 11-week-old female SD rats (weighing 230–260 g) and 15 age-matched male rats were purchased from Liaoning Changsheng Biotechnology Co., Ltd. [Liaoning, China; license No. SCXK (Liao) 2020−0001]. The animals were maintained in conventional laboratory settings (20 ± 3 °C, 45 ± 5% relative humidity, 12/12-hour light/dark cycle) with ad libitum access to chow and drinking water. All experimental procedures were reviewed and authorized by the Ethics Committee of Henan University of Chinese Medicine (Henan, China) in accordance with the National Institutes of Health (NIH) standards for the ethical care and utilization of laboratory animals.

### Animal Treatment

#### Mating and Prenatal Fear Stress Procedure

All female rats and male rats were mated in a cage in the ratio of 2:1 according to our previous study (Li et al. 2024). Successful copulation and gestation were confirmed by the detection of vaginal spermatozoa in female rats. After conception, 30 pregnant rats were randomly assigned to two groups: the normal control (NC) group and fear stress model (FSM) group (*n* = 15 per group). The prenatal fear stress procedure was performed as previously described. Briefly, pregnant rats assigned to the FSM group were positioned in a modified electrical shock chamber to observe and audibly perceive the distress of their male partners subjected to electrical stimulation over 20 consecutive days. In contrast, pregnant rats in the NC group were placed in the same environment, but they were not exposed to psychological fear stress because their male partners did not receive electrical stimulation. Specifically, the apparatus remained inactive (no electrical stimulation applied to the chamber floor), ensuring male rats exhibited no aversive behaviors (e.g., distress vocalizations or jumping) that could convey fear cues to the pregnant females. Additionally, during this time, all pregnant females were housed individually, separated from male rats.

#### Experimental Procedures and Sample Preparation

To ensure the stability of the model, we assessed the behavioral responses of pregnant rats and measured the levels of stress‑related hormones to confirm their stress status. On gestational day 17, six fasted (fasted for 12 h) pregnant rats per group were exposed to 30 min of fear stress, and then were promptly anesthetized via intraperitoneal administration of sodium pentobarbital (50 mg/kg). Blood samples were collected via abdominal aorta puncture within 2 h after stress exposure. Serum samples were separated and preserved at −80 °C for subsequent analysis. After blood collection, we performed a cesarean section, recorded litter size and individual pup masses, and then immediately collected the placentas and pup brains. Subsequently, the hypothalamus of the pregnant rats was separated. All tissues were stored in a −80 °C freezer for subsequent experiments. All biochemical analyses were completed within three months after sample collection.

Remaining pregnant rats (7 and 5 rats in the NC and FSM group, respectively) were subjected to scheduled fear stress daily until spontaneous parturition. Offspring physical development was assessed by recording litter size, pup survival rate, and body weight, while somatic developmental milestones were evaluated following established protocols (Chiavegatto et al. 1997; Smart and Dobbing 1971; Chen et al. 2012; Ivani et al. 2016), with daily monitoring continued until all pups achieved each milestone.

Postnatal day 21 (PND21) in offspring rats represents a critical developmental juncture that coincides with weaning, the slowdown of hippocampal synaptogenesis, and the functional maturation of the hippocampal memory system, and is also a well‑established time point for assessment in early life stress research (Lauterborn et al. 2017). On PND 21, 10 pups were randomly selected for behavioral assessment (at least one male or female offspring per litter). Pups were first subjected to 30 min of acute restraint stress, followed sequentially by the open field test (OFT), tail suspension test (TST), and irritability scoring. The sucrose preference test (SPT) was performed on days 22–24, and the Morris water maze (MWM) test on days 24–28. After completion of all behavioral assessments, the same 10 pups received another 30 min of acute restraint stress, followed immediately by blood collection and brain removal to isolate the hypothalamus and hippocampus.

The remaining pups were anesthetized with 2% pentobarbital sodium (w/v, 50 mg/kg), and abdominal aortic blood was collected for serum isolation. For each group, 4 whole-brain samples were fixed in 4% paraformaldehyde for histopathological examination; another 4 hippocampal samples were cut into 1 mm × 1 mm pieces and immersed in glutaraldehyde solution for electron microscopy. The hypothalamic and hippocampal tissues of the remaining pups were rapidly dissected and stored at −80 °C for subsequent WB, qPCR and ELISA assays.

### Method of Behavioral Testing

#### SPT

The SPT (Wu et al. 2016) was performed as follows: initially, 2 days before the formal test, rats were acclimated to sucrose solution by providing one bottle of 1% sucrose solution and one bottle of tap water in each cage randomly. The positions of the two bottles were exchanged after 12 h to facilitate acclimation. Following the acclimation period, rats were deprived of food and water for 24 h. Subsequently, each rat was given free access to one bottle of 1% sucrose solution and one bottle of tap water. To control for potential side preference, the positions of the two bottles were switched after the third hour. Liquid consumption was measured after a total testing period of 6 h. Sucrose preference was computed via the following equation: (sucrose intake)/(sucrose intake + water intake) × 100%.

#### OFT

The OFT (Colla et al. 2015) was conducted as follows: Rats were placed in the central zone of an open-field arena, which measured 100 centimeters in length, 100 centimeters in width, and 50 centimeters in height. To standardize the environmental conditions and elicit anxiety-related exploratory behaviors, the illumination level was set to 250–300 lx in the central zone of the arena and 60–80 lx in the peripheral zones. The base of the device was segmented into twenty-five congruent compartments. The rats’ movement trajectories were captured using a video camera over a 5-minute observation period. To ensure hygiene and prevent cross-contamination between trials, the apparatus was thoroughly sanitized with alcohol after each experimental run.

#### Irritability Scores

Irritability scores for each rat were documented following the open-field experiment in a blind-controlled manner, utilizing a four-point scale methodology as outlined in reference (Li et al. 2022). The following grading criteria were employed: Grade 4 was assigned when the rat emitted high-pitched screams, exhibited jumping behavior, attempted to bite the person upon tail lifting, gnawed on the cage, or frequently engaged in aggressive interactions with cage-mates; Grade 3 was designated for rats that readily displayed biting tendencies when gently restrained by the scruff of the neck; Grade 2 was noted for rats that emitted screams and displayed startle responses upon being restrained via neck capture; and Grade 1 was allocated when none of the aforementioned behaviors were conspicuously observed.

#### TST

The TST was performed as previously described (Wu et al. 2016). Animals were individually suspended by their tails (at a height of 60 cm from the ground) for 6-minute duration. Immobility time (defined as passive hanging without escape-oriented movements) was automatically recorded during the final 4-minute interval using video tracking software.

#### MWM

The MWM test was conducted following previously described protocols (Wang et al. 2017): using circular black pool (100 cm diameter × 60 cm height) containing opaque water (25–27 °C; rendered opaque with non-toxic white paint) and a submerged platform (1 cm below water surface). The experiment consisted of two phases: (1) a 4-day place navigation (preliminary training) phase: the platform was positioned at the center of the northern quadrant, individual rats were placed in the water facing the pool wall, at one of four semi-randomly assigned starting points (north, south, east, and west) and given 120 s to find the submerged platform. Rats that located the platform on their own remained there for 5 s; those that didn’t were gently maneuvered to the platform and detained there for 30 s. Each rat completed four training sessions daily across four successive days, with escape latency documented. (2) Following the 4-day instructional period, an assessment of mnemonic retention was performed via spatial probe examination. On the fifth day, the platform was withdrawn, and rats were placed into the pool from the quadrant contrary to its prior location, with a 90-second search window. A video-tracking system (CG-400 Image Acquisition System, Institute of Materia Medica, Chinese Academy of Medical Sciences, Shanghai) meticulously recorded and analyzed crossing frequency over the original platform position, swimming velocities, and time elapsed in the target quadrant. Following the trial, rats were dried, kept warm, and housed back in their enclosures.

### Biochemical Assay

The CRF(cat. no. 0520R1) levels in the hypothalamus and placenta, serum ACTH, (cat. no. 0565R1), and CORT(cat. no. 0559R1) levels, and hippocampal IP3 (cat. no. 21020R1) levels were measured by enzyme-linked immunosorbent assay (Jiangsu Enzyme Exemption Industry Co., Ltd, Jiangsu, China).

### Total RNA Isolation and qPCR

The hippocampal samples were subjected to RNA extraction and purification employing TRIzol reagent (Cat No. G3013, Servicebio, Wuhan, China). RNA concentration was assessed using a Nanodrop 2000 spectrophotometer (Thermo Fisher Scientific, MA, USA). Subsequently, RNA samples were reverse-transcribed into single-stranded cDNA using a Synthesis SuperMix (One-Step gDNA Remover, Servicebio, Wuhan, China). qPCR was performed using 2×SYBR Green qPCR Master Mix (None ROX, Servicebio, Wuhan, China). The specific qPCR primers were designed and synthesized by Wuhan Servicebio Technology Co., Ltd. (Wuhan, China), and their sequences are listed in Table 1. The relative mRNA expression levels of target genes were calculated using the 2^−∆∆CT^ method and normalized to GAPDH.

**Table 1:** Rat qPCR primer sequences

Primer name	Primer sequence(5’−3’)
R-GAPDH- F	CTGGAGAAACCTGCCAAGTATG
R-GAPDH- R	GGTGGAAGAATGGGAGTTGCT
R-CRHR1- F	CCGCTACAACACGACAAACAA
R-CRHR1- R	GTAGGATGAAAGCCGAGATGAGG
R-CRHR2- F	AAAACTGCGAGCCTCCACC
R-CRHR2- R	AAAGAAACCCTGAAAGGACTGC
R-Plcb1- F	AGTTGGCTGGGAACTCGTCTG
R-Plcb1- R	ACACTCTGCGATGGCTTCTATG
R- IP3R1- F	CGAATGGATTTATCAGCACCTT
R- IP3R1- R	ACCGCATCTGTTGTACTGTTGG
R- VDAC1-F	TTCCAGCTTCATACTAATGTGAATG
R- VDAC1-R	CTAAGCCAATTAGACTGGAGTTGTT

### Western Blot Analysis

Total proteins were extracted from the hippocampal tissues using RIPA lysis buffer, which consisted of 50 mM Tris (pH 7.4), 150 mM NaCl, and 1% Triton X-100, 1% sodium deoxycholate, and 0.1% SDS, supplemented with PMSF, protease inhibitors, and phosphatase inhibitor. Protein concentration was determined using a BCA protein assay kit (Jiangsu Meimian Industrial Co., Ltd., Jiangsu, China). Subsequently, protein samples were separated by SDS-PAGE and transferred onto PVDF membranes using a Bio-Rad electrophoresis system (1645050, USA). After blocking with 5% (w/v) non-fat milk for 2 h, the membranes were incubated overnight at 4 °C with the primary antibodies against: CRF receptor 1 (CRFR1, 1:1000, Cat. No. GB11707-100, Servicebio, Wuhan, China), CRFR2 (1:1500, Cat. No. GB11709-100, Servicebio, Wuhan, China), Inositol 1,4,5‑trisphosphate receptor type 1 (IP3R1, 1:500, Cat. No. GB11742-100, Servicebio, Wuhan, China), Phospholipase C beta 1 (PLCβ1, 1:1000, Cat. No. 26551-1-AP, Proteintech, Wuhan, China), Voltage‑dependent anion channel 1 (VDAC1, 1:1000, Cat. No. GB111939-100, Servicebio, Wuhan, China), Mitochondrial Calcium Uniporter (MCU, 1:800, Cat. No. GB111353-100, Servicebio, Wuhan, China) and α-tubulin (1:4000, Cat. No. GB15201-100, Servicebio, Wuhan, China). After three washes with 1× TBST (Servicebio, Wuhan, China), the membranes were incubated with HRP-conjugated secondary antibodies (1:20000, Servicebio, Wuhan, China). Protein bands were visualized using a Thermo Fisher gel imaging system (MA, USA) and the band intensities were quantified using ImageJ software. α-tubulin was used as an internal control to normalize protein loading.

### Histological Examination

Brain tissues were dehydrated in a graded ethanol series (80%, 95%, and 100%) and embedded in paraffin. Subsequently, serial coronal sections of 4‑µm thickness were prepared, and six sections were collected from each sample. These sections were used for hematoxylin and eosin (H&E) staining (Servicebio, Wuhan, China) and subsequent immunofluorescence staining.

### Immunofluorescence Combined TUNEL Staining

Hippocampal tissue sections were processed for immunofluorescence combined TUNEL staining (G1501, Servicebio, Wuhan, P.R. China). Briefly, the sections were deparaffinized with xylene, rehydrated through a graded ethanol series, and permeabilized with Proteinase K. Sections were incubated with 100 µL TDT equilibration buffer at 37 °C for 30 min, followed by incubation with 50 µL TUNEL working solution in a humidified chamber at 37 °C for 70 min. The sections were then blocked with 3% BSA at room temperature for 30 min, followed by incubation with primary antibody against NeuN (1:200, GB11138, Servicebio, China) overnight at 4 °C. After washing, the sections were incubated with fluorescence-conjugated secondary antibody at room temperature for 1 h. Following three washes with PBS, nuclei were counterstained with DAPI. Sections were mounted with antifade mounting medium, and images were captured using an orthogonal fluorescence microscope (Nikon Eclipse C1, Japan).

### Immunofluorescent Staining

Briefly, 5-µm-thick paraffin-embedded coronal sections were subjected to deparaffinization and rehydration procedures. Antigen retrieval was accomplished by boiling the sections in 0.01 M citrate buffer (pH 6.0) for 10 min. For double - staining of GRP75 and VDAC1, brain sections were incubated with primary antibodies against GRP75 (1:600, Servicebio, GB112273) and VDAC1 (1:500, Servicebio, GB121939) at 4 °C overnight. Subsequently, the sections were incubated with corresponding secondary antibodies for 2 h at room temperature: anti-rabbit IgG Alexa Fluor 488 (1:500, Servicebio, GB25303) and anti-mouse IgG Alexa Fluor 594 (1:500, Servicebio, GB28303). The sections were then sealed with ProLong^®^ Gold Antifade Reagent containing DAPI (CST, #8961). Images were captured using an orthogonal fluorescence microscope (Nikon Eclipse C1, Japan).

### Electron Microscopy

Transmission electron microscopy (TEM) was performed at the Electron Microscope Center of the Scientific Research and Experiment Center, Henan University of Chinese Medicine. Hippocampal samples were fixed in 2.5% glutaraldehyde for 4 h, followed by four washes with 0.1 M PBS (15 min each). Subsequently, samples were post-fixed in 1% osmium tetroxide for 1.5 h and rinsed 3 times with PBS. The specimens were dehydrated in a graded ethanol series (50%, 70%, 80%, 90%, and 100%), and then infiltrated and embedded in epoxy resin 812: sequentially in a 1:1 mixture of resin and acetone, a 2:1 mixture, and finally pure resin, followed by curing overnight at room temperature. Ultrathin Sects. (50–60 nm) were cut using an ultramicrotome, stained with uranyl acetate for 20 min, and observed by TEM at 40,000–80,000× magnification.

### Cellular Experiments

#### Cell Culture

SH-SY5Y human neuroblastoma cells were cultured in Dulbecco’s Modified Eagle Medium/Nutrient Mixture F-12 (DMEM/F12) supplemented with 10% fetal bovine serum (FBS) and 1% penicillin–streptomycin. Cells were maintained in a humidified incubator at 37 ℃ with 5% CO₂. The culture medium was replaced every 2–3 days, and subculture was performed when cell confluence reached 70%–80%. Before subculture, cells were washed twice with PBS, digested with prewarmed 0.25% trypsin-EDTA, and the digestion was terminated using complete DMEM/F12 medium (containing 10% FBS and 1% penicillin–streptomycin). After centrifugation at 1000 rpm for 5 min at room temperature, the supernatant was discarded, and the cell pellet was resuspended in 1 mL fresh complete medium. The cell suspension was diluted 10-fold, and 10 µL was loaded into a cell counting chamber. Cell counting was performed under an inverted microscope, and cells were adjusted to a density of 1 × 10⁴ cells/mL for subsequent assays in 96-well plates.

#### Evaluation of Cell Viability Following CRH Overexposure in SH-SY5Y Cells and Effects of CP376395 or DS16570511

SH-SY5Y cells in the logarithmic growth phase were seeded into 96-well plates at 1.0 × 10⁴ cells/well and incubated overnight at 37 ℃ in a 5% CO₂ humidified incubator. To establish a chronic stress-like cell model, cells were exposed to various concentrations of CRH (0.4, 2, 5, 10, 20, µM) for different durations (48 h and 96 h) to evaluate the effects of CRH concentration and exposure time on cell viability. In addition, to investigate the protective effect of the CRH receptor 1(CRHR1) specific inhibitor CP376395, cells were co‑incubated with 0.1 µM CP376395 for 48 h. To further determine whether CRH overexposure‑induced proliferation inhibition is associated with mitochondrial calcium overload, rescue experiments were performed using the MCU inhibitor DS16570511. Cells were first treated with CRH for 48 h to establish the inhibition model, followed by co‑treatment with DS16570511 and CRH for additional 12 h. Cell viability was detected using the Cell Counting Kit-8 (CCK-8) assay.

#### Establishment of SH‑SY5Y Cell Lines with Stable PLCβ1 Knockdown

Stable SH‑SY5Y cell lines with PLCβ1 knockdown (PLCβ1‑KD) were constructed using lentiviral vector transfection. The interference sequence was PLCβ1‑sh: CAGCGAGATCCTCGGCTTAAT, and the negative control (NC‑KD) sequence was CCTAAGGTTAAGTCGCCCTCG. All plasmids were constructed by Chongqing Youbao Biotechnology. Recombinant plasmids and packaging plasmids were co‑transfected into 293FT cells for lentivirus packaging. The viral supernatants were harvested, filtered, aliquoted, and stored at ‑80 ℃. SH‑SY5Y cells at 70% confluence were infected with the lentiviruses, and polybrene was added to enhance infection efficiency. The optimal screening concentration of puromycin was determined by concentration gradient assay, and infected cells were selected to obtain stable PLCβ1‑KD and NC-KD cell lines, which were expanded for subsequent experiments.

#### Effect of PLCβ1 Knockdown on CRH‑Induced Cell Injury Detected by CCK‑8 Assay

SH‑SY5Y, NC-KD, and PLCβ1‑KD cells were seeded into 96‑well plates at 5000 cells per well and incubated overnight for attachment. Cells were divided into a control group, a NC-KD group, a PLCβ1‑KD group, and PLCβ1‑KD groups treated with different concentrations of CRH (5, 10, and 20 µM). After 96 h of CRH treatment, cell viability was detected using the CCK‑8 assay to analyze the effect of PLCβ1 knockdown on CRH‑induced injury in SH‑SY5Y cells.

#### Extraction of Total Protein and Total RNA from Cell Samples

SH-SY5Y cells, NC-KD, and PLCβ1‑KD SH‑SY5Y cells were treated as described above (the cell confluence of the control group was approximately 90%). After discarding the culture medium, the cells were washed 2–3 times with pre-chilled PBS, harvested, and centrifuged at 12,000 rpm for 5 min at 4 ℃ to obtain the cell pellet. The procedures for total protein extraction, denaturation, and storage were consistent with those described in the aforementioned animal experiments; the extraction of total RNA, concentration detection, reverse transcription, as well as the reaction system and amplification conditions for subsequent real-time quantitative PCR (RT-qPCR) were all consistent with the aforementioned animal experimental methods (see Tables 2 and 3).

**Table 2:** SH-SY5Y cells qPCR primer sequences

Primer name	Primer sequence(5’−3’)
hGAPDH-F	CCATGGGTGGAATCATATTGGA
hGAPDH-R	TCAACGGATTTGGTCGTATTGG
hPLCβ1-F	GGAAGCGGCAAAAAGAAGCTC
hPLCβ1-R	CGTCGTCGTCACTTTCCGT

**Table 3:** PCR amplification conditions

Steps	Temperature	Duration	Cycle number
Initial denaturation	95℃	3 min	–
Cycling reactions	95℃	5 s	40
	60℃	≥ 20 s	
Melting curve	95℃	15 s	–
	60℃	60 s	
	95℃	15 s	

#### Mitochondrial Calcium Detection

SH-SY5Y cells, NC-KD, and PLCβ1‑KD SH‑SY5Y cells were seeded into 96-well plates at 1 × 10^4^ cells per well. After treatment as described above, the culture medium was removed, and the cells were gently washed with pre-warmed PBS. Cells were incubated with 100 µL Rhod-2 AM staining solution per well in the dark for 30 min, washed with PBS, and then subjected to fluorescence intensity detection of mitochondrial calcium ions using a Cell Imaging Multi-Mode Microplate Reader (Agilent BioTek Cytation 5) at excitation and emission wavelengths of 552 nm and 581 nm, respectively. Representative images were captured using a fluorescence microscope.

### Statistical Analyses

The results are represented by means ± SD and analyzed using GraphPad Prism 10.0. Comparisons between two groups used Student’s t‑test (or Welch’s t‑test for unequal variances); multiple groups used ANOVA with Tukey’s post hoc test. Non‑normally distributed data were analyzed by Mann‑Whitney U test (Holm‑Šídák correction). Two‑way ANOVA (group × sex) and two‑way repeated measures ANOVA (escape latency) were applied. Developmental milestone attainment times were compared by log‑rank test. *p* < 0.05 was considered significant.

## Results

### Bioinformatics Analysis

**Fig. 1:**
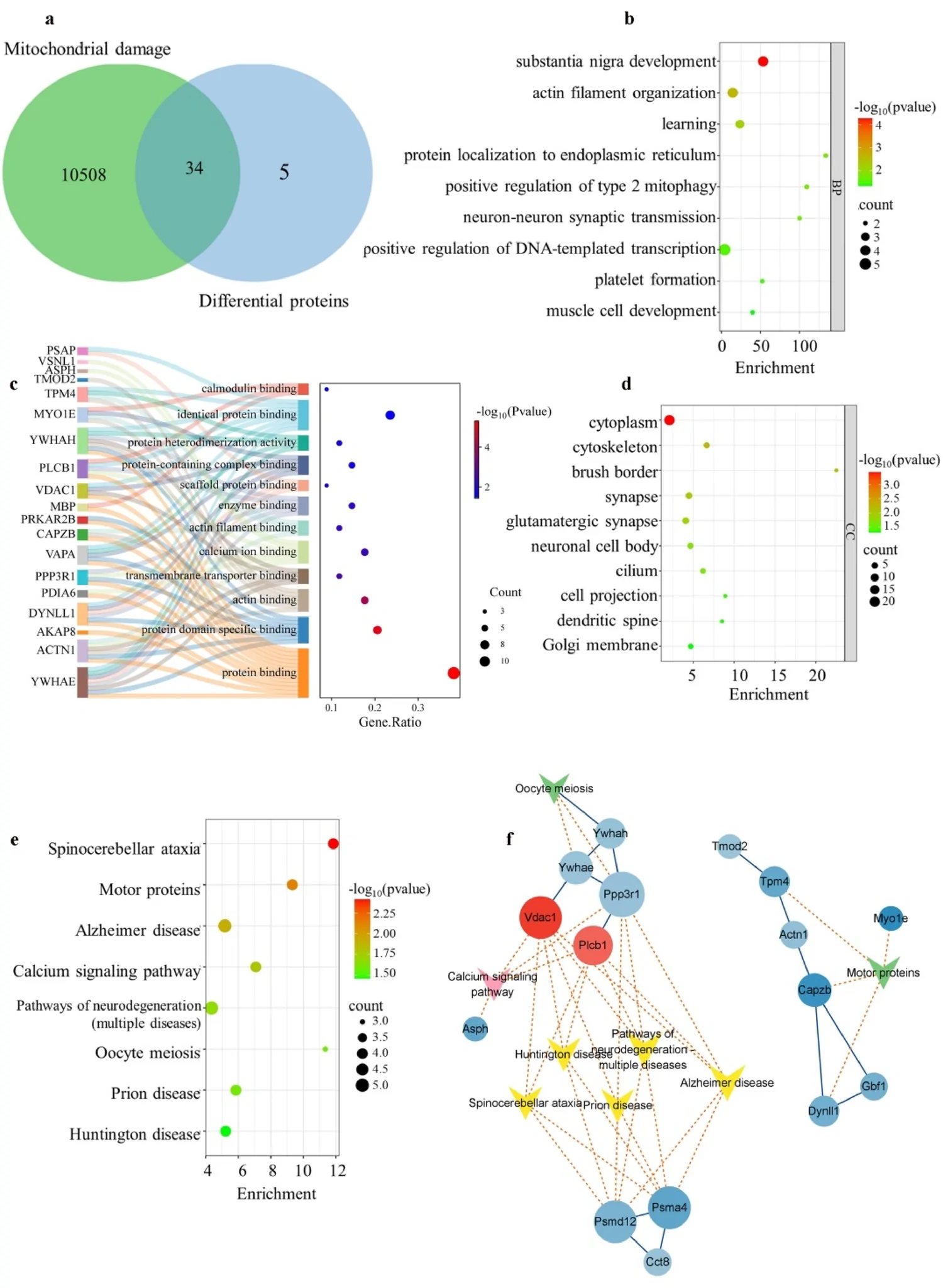
GO, KEGG enrichment analyses and PPI network of overlapping proteins. **a** Venn diagram of overlapping proteins. **b**–**d** the GO classification of overlapping proteins (p-value < 0.05). **e** The pathways with substantial enrichment (p-value < 0.05). **f** Pathway-PPI association network (p-value < 0.05); Circle-nodes denote overlapping proteins; Arrow-shaped nodes signify the pathways; Blue solid edges indicate strong protein–protein interaction, while orange dashed edges represent proteins enriched in corresponding pathways

Using “mitochondrial damage” as keyword, 10,539 targets were retrieved from GeneCards (relevance score > 1) and 310 from NCBI, yielding 10,542 unique targets after merging. These were intersected with DEPs (fold change > 1.5 or < 0.67, *P* ≤ 0.05), resulting in 34 overlapping proteins as candidate targets of prenatal stress-induced mitochondrial damage (Fig. 1a, and Tables 4 and 5). These 34 targets were subjected to GO and KEGG enrichment analyses to explore the underlying mechanisms.

**Table 4:** Unregulated overlapping proteins

Gene Names	Protein Names
Mbp	Isoform 1 of Myelin basic protein S
Plcb1	1-phosphatidylinositol-4,5-bisphosphate phosphodiesterase beta-1
Pdia6	protein disulfide-isomerase A6
Fubp3	MAP2 RNA trans-acting protein 2
Fubp1	Far upstream element-binding protein 1
Nap1l4	Nucleosome assembly protein 1-like 4
Vdac1	Voltage-dependent anion-selective channel protein 1
Akap8	A-kinase anchor protein 8
Pmpcb	Mitochondrial-processing peptidase subunit beta

**Table 5:** Downregulated overlapping proteins

Gene Names	Protein Names
Vapa	Vesicle-associated membrane protein-associated protein A
Myo1e	Myosin-Ie
Capzb	F-actin-capping protein subunit beta
Psap	Sulfated glycoprotein 1
Astn1	astrotactin 1
Tomm7	translocase of outer mitochondrial membrane 7 homolog
Psma4	Proteasome subunit alpha type-4
Arl3	ADP-ribosylation factor-like protein 3
Asph	Aspartyl/asparaginyl beta-hydroxylase
Tpm4	Tropomyosin alpha-4 chain
Myof	myoferlin-like
Dynll1	Dynein light chain 1, cytoplasmic
Gbf1	Golgi-specific brefeldin A-resistance guanine nucleotide exchange factor 1
Psmd12	Proteasome (Prosome, macropain) 26 S subunit, non-ATPase, 12
Lap3	Isoform 1 of Cytosol aminopeptidase
Zdhhc15	Palmitoyltransferase ZDHHC15
Prkar2b	cAMP-dependent protein kinase type II-beta regulatory subunit
Actn1	Alpha-actinin-1
Vsnl1	Visinin-like protein 1
Ppp3r1	Isoform 2 of Calcineurin subunit B type 1
Ywhah	14-3-3 protein eta
Atp6v1f	V-type proton ATPase subunit F
Cct8	T-complex protein 1 subunit theta
Ywhae	14-3-3 protein epsilon
Tmod2	Tropomodulin-2

To delineate the potential mechanism of prenatal stress-induced mitochondrial damage, we analyzed the 34 overlapping proteins in depth. Biological process enrichment highlighted protein localization to endoplasmic reticulum, positive regulation of type 2 mitophagy, and neuron-neuron synaptic transmission (Fig. 1b). Molecular function analysis revealed calcium ion binding, and calmodulin binding activities (Fig. 1c). Cellular component annotation indicated significant enrichment at the synapse, neuronal cell body, dendritic spine, and Golgi membrane (Fig. 1d). Pathway analysis identified Calcium signaling pathway, and multiple neurodegenerative disease signaling pathways (Fig. 1e). The PPI network of overlapping proteins displayed three main subnetworks. To delineate the role of overlapping proteins in mitochondrial damage, an integrated network combining pathway enrichment and PPI data was constructed using Cytoscape software (Fig. 1f). Arrow-shaped network nodes represent signaling pathways, while orange dashed edges represent proteins enriched in corresponding pathways. Notably, within the two disease-associated subnetworks, key proteins (PLCβ1, VDAC1, and PPP3R1) participating in the calcium signaling pathway were also simultaneously linked to the neurodegenerative disease network, suggesting that calcium dysregulation may serve as a molecular bridge connecting mitochondrial damage to neurodegeneration. In addition, both PLCβ1 and VDAC1 are significantly upregulated proteins. The overactivation of these two proteins may lead to mitochondrial calcium overload and eventually result in apoptosis (Yu et al. 2021). Collectively, these bioinformatic analyses revealed that the calcium signaling‑related proteins PLCβ1, VDAC1, and PPP3R1 are concurrently enriched in the neurodegenerative disease network, suggesting that calcium signaling abnormalities may serve as a potential molecular bridge linking mitochondrial damage to neurodegeneration. Notably, both PLCβ1 and VDAC1 are upregulated proteins, and their overactivation may be involved in mitochondrial calcium overload.

### In Vivo Validation of the Candidate Pathway in Prenatally Stressed Offspring

#### Effects of Prenatal Stress on Maternal Behavior, HPA Axis Activity, and Adverse Litter Outcomes

**Fig. 2:**
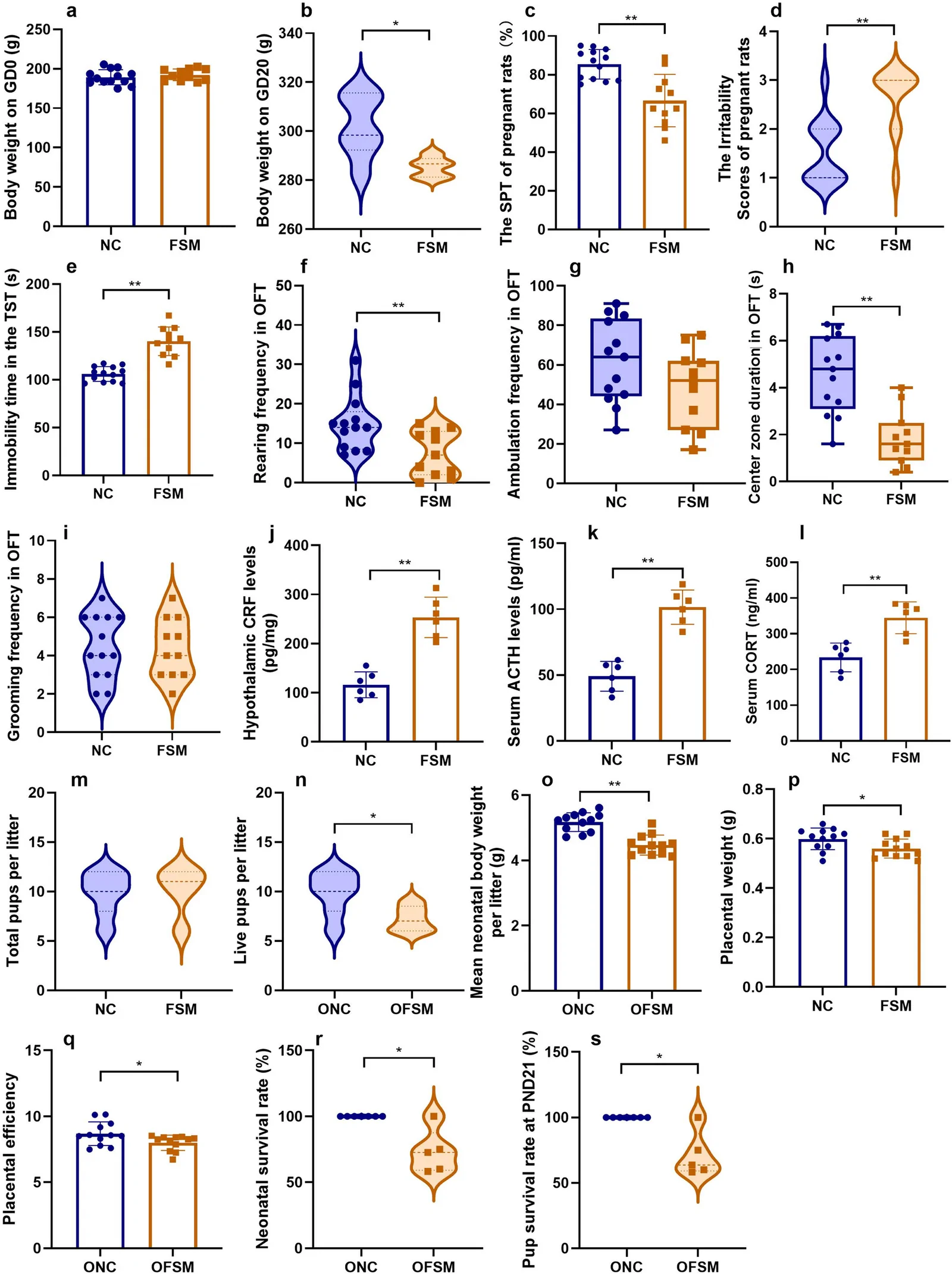
Effects of prenatal stress on maternal behavior, HPA axis activity, and adverse litter outcomes. **a** and **b** Body weight of rats at pre-pregnancy and late gestation was recorded. **c**–**i** Behavioral assessment results of pregnant rats were recorded. **j**–**l** HPA axis-related hormone levels in pregnant rats were measured. **m**–**s** Effects of prenatal stress on litter outcomes evaluated. NC, normal control group; FSM, fear stress model group; ONC, offspring of normal control group; OFSM, offspring of fear stress model group; Continuous variables with equal variances were analyzed by Student’s t-test, and those with unequal variances by Welch’s t-test. Non-normal data (grooming frequency, irritability scores) were analyzed by Mann-Whitney U test. *p < 0.05, **p < 0.01

As presented in Fig. 2a and b, body weights of dams in the two groups were similar before exposure to fear stress. Nevertheless, over the 20-day fear stress period, the weight gain rate of pregnant rats in the FSM group was significant decreased (*p* < 0.05). In addition, compared with the NC group, the FSM group exhibited a significantly decreased sucrose preference (Fig. 2c), prolonged immobility time in the TST (Fig. 2e), and increased irritability scores (Fig. 2d). Moreover, the FSM group showed reduced rearing frequency (Fig. 2f) and center zone duration (Fig. 2h) in the OFT. Furthermore, the FSM group showed a hyperactive HPA axis, as indicated by significantly increased hypothalamic CRH, serum ACTH, and CORT levels (Fig. 2j, k, l). These results suggest that psychological fear stress leads to increased stress hormone levels and behavioral impairments in pregnant rats.

To evaluate the effects of prenatal stress on litter characteristics, we measured parturition-related parameters, placental metrics, and offspring survival. The results showed that the FSM group had significantly lower live births (Fig. 2n), neonatal pup weight (Fig. 2o), placental weight (Fig. 2p), placental efficiency (Fig. 2q), and survival rates on PND4 and PND21 (Fig. 2r and s) compared with the control group.

#### Behavioral Assessment of Pups

**Fig. 3:**
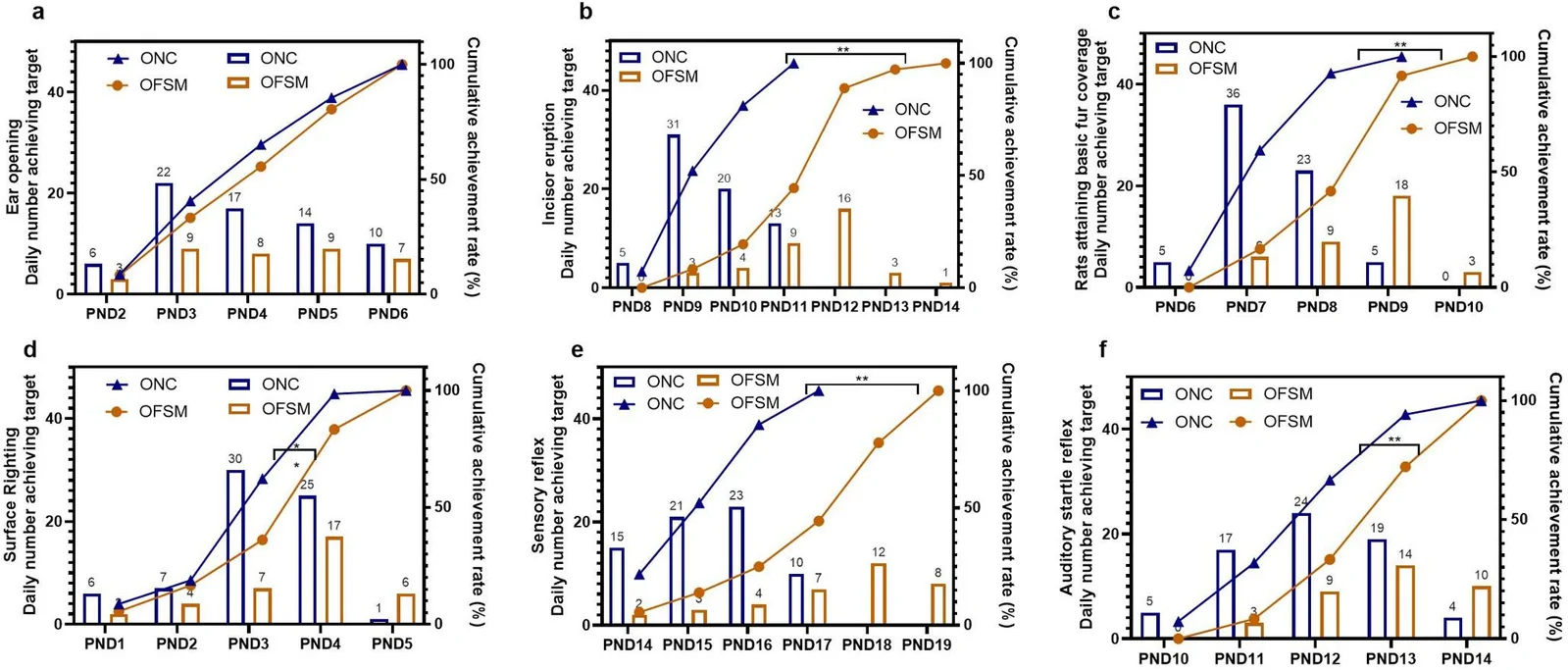
Attainment times of developmental and reflex milestones in pups. The results of (a) Ear opening, (b) Incisor eruption, (c) Rats attaining basic fur coverage, (d) Surface Righting, (e) Sensory reflex, and (f) Auditory startle reflex were recorded. Statistical significance was determined using the log-rank test. *p<0.05, **p<0.01.

To ensure the stability and reliability of the methodology, developmental milestones and behavioral assessments were conducted on pups. As shown in Fig. 3, Compared with the offspring from normal control group NC group (ONC), the offspring from FSM group (OFSM) showed significantly prolonged attainment times for developmental and reflex milestones, including incisor eruption, full fur coverage, surface righting reflex, sensory reflex, and auditory startle reflex.

**Fig. 4:**
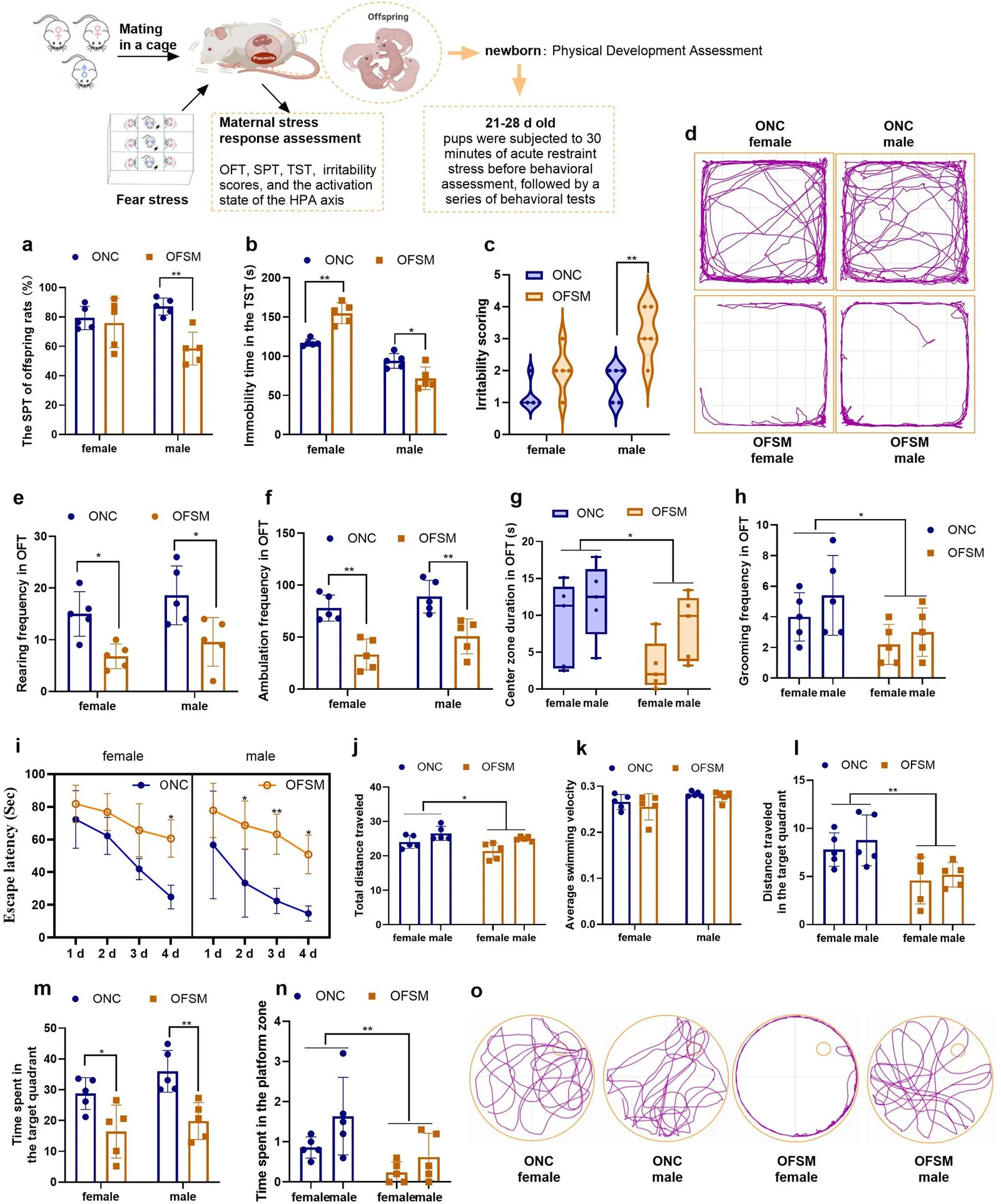
Behavioral assessment of pups. The results of SPT (**a**), immobility time in TST (**b**), and irritability scores (**c**) of pups were recorded; **d**–**h** the results of Open field test were recorded; **i**–**o** Learning and memory abilities of the pups were evaluated using the MWM. Escape latency was analyzed by two‑way repeated measures ANOVA. Irritability scores were compared using multiple Mann‑Whitney U tests with Holm-Šídák correction. Other data were analyzed by two‑way ANOVA (group × sex) with Tukey’s post hoc test. *p* < 0.05p < 0.05 was considered significant. *p < 0.05, **p < 0.01

Behavioral assessments revealed that, compared with ONC group, OFSM group male pups had significantly lower sucrose preference (Fig. 4a), females had longer tail suspension immobility but males had shorter immobility, and males had higher irritability scores (Fig. 4b and c). In the OFT (Fig. 4d and h), both sexes showed reduced rearing, ambulation, grooming and center zone duration. In the MWM test (Fig. 4i and o), OFSM males showed longer escape latencies on days 2–4, while females showed shorter latency only on day 4. In the probe trial, model pups of both sexes showed decreased swimming distances, platform zone search time, and target quadrant time. Our findings showed that prenatal fear stress exposure might lead to elevated anxiety- and depression-like behaviors, as well as impaired spatial learning and memory abilities in offspring.

#### Effects of Prenatal Stress on Offspring HPA Axis Function

**Fig. 5:**
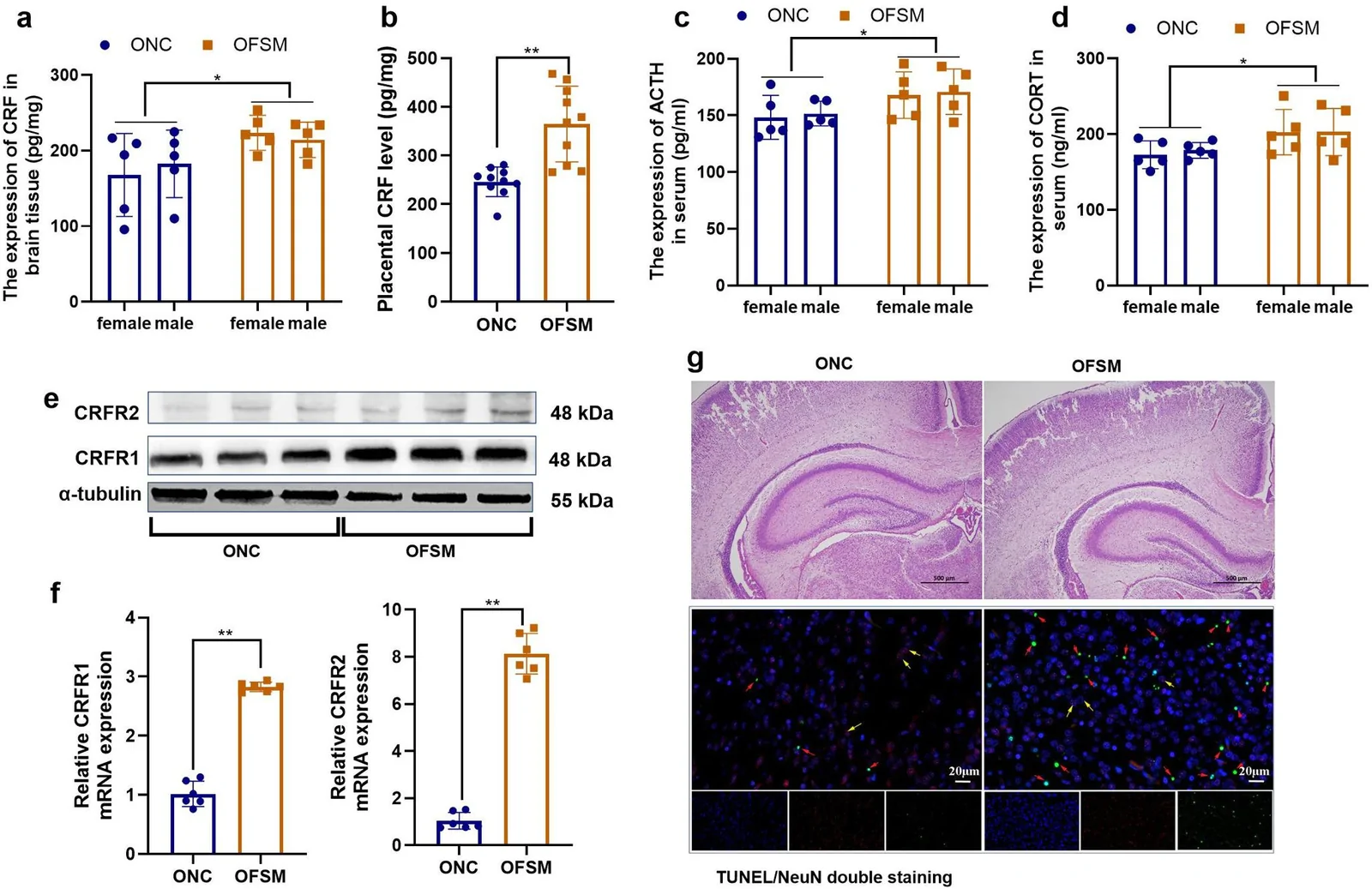
Effects of prenatal stress on neuroendocrine and brain tissue damage in neonatal pups. The levels of CRF in brain (**a**) and placenta (**b**), serum ACTH (**c**) and corticosterone (**d**) were determined; the protein (**e**) and mRNA (**f**) expressions of CRHR1 and CRHR2 in brain tissue were determined; **g** Representative HE-stained brain sections and representative immunofluorescence/TUNEL images. Statistical significance of mRNA expression was determined using Student’s t-test, and that of other data using two-way ANOVA. *p < 0.05, **p < 0.01 vs. control group

OFSM group neonatal pups exhibited HPA axis activation, as evidenced by elevated CRF levels in brain (Fig. 5a) and placenta (Fig. 5b), increased serum ACTH (Fig. 5c) and CORT (Fig. 5d), and upregulated brain CRF receptor 1/2 mRNA (Fig. 5f) and protein (Fig. 5e) expression. In addition, OFSM neonatal pups showed significantly increased neuronal apoptosis in the brain, as detected by immunofluorescence/TUNEL staining (Fig. 5g).

**Fig. 6:**
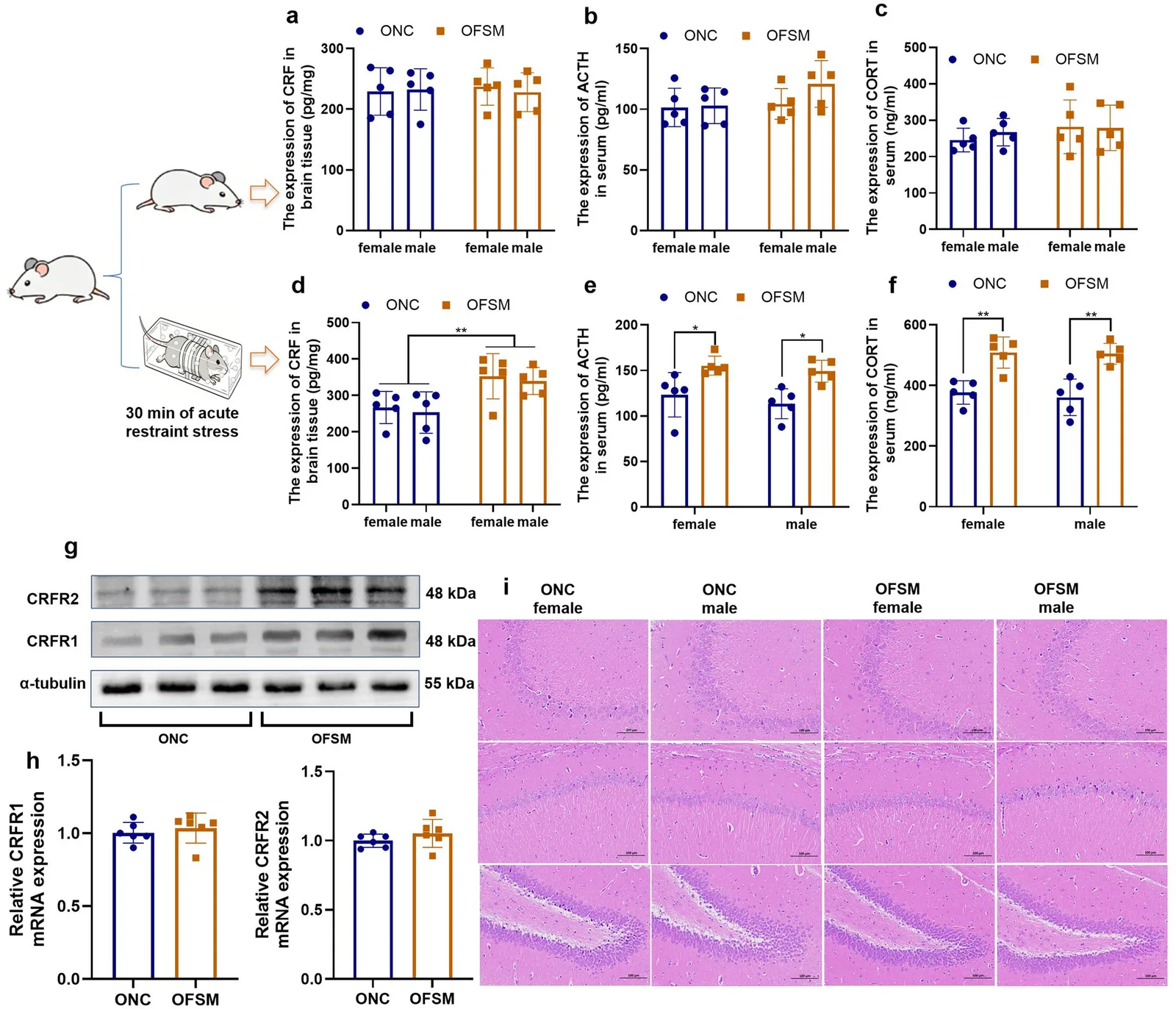
HPA axis function, receptor expression, and brain morphology in early‑socialization‑stage pups. **a**–**c** The levels of Hypothalamic CRF, serum ACTH and corticosterone under basal conditions were determined; **d**–**f** The same hormones after acute restraint stress were determined; **g** Representative western blots of hippocampal CRFR1 and CRFR2 protein; **h** Relative CRFR1 and CRFR2 mRNA expression were determined. **i** Brain HE staining (scale bar = 100 μm). Statistical significance of mRNA expression was determined using Student’s t-test, and that of other data using two-way ANOVA. *p < 0.05, **p < 0.01 vs. control group

The examination results of pups at the early socialization stage (PND 21–30) showed that basal hypothalamic CRF and serum ACTH/corticosterone levels did not differ between groups (Fig. 6a–c). However, following 30-min acute restraint stress, the OFSM group exhibited significantly higher levels of all three hormones, indicating latent HPA axis hyper-reactivity (Fig. 6d–f). In addition, the OFSM group showed significantly upregulated hippocampal CRFR1/CRFR2 protein and mRNA (Fig. 6g, h). PND21 brain HE staining was unremarkable (Fig. 6i), whereas apoptotic cells were observed in neonates (Fig. 5g), revealing a pattern of “resolved early damage with persistent receptor abnormalities.”

#### Prenatal Stress Induces Structural Alterations in MAMs and Enhanced IP3 Responses to Acute Stress in Offspring Hippocampus

**Fig. 7:**
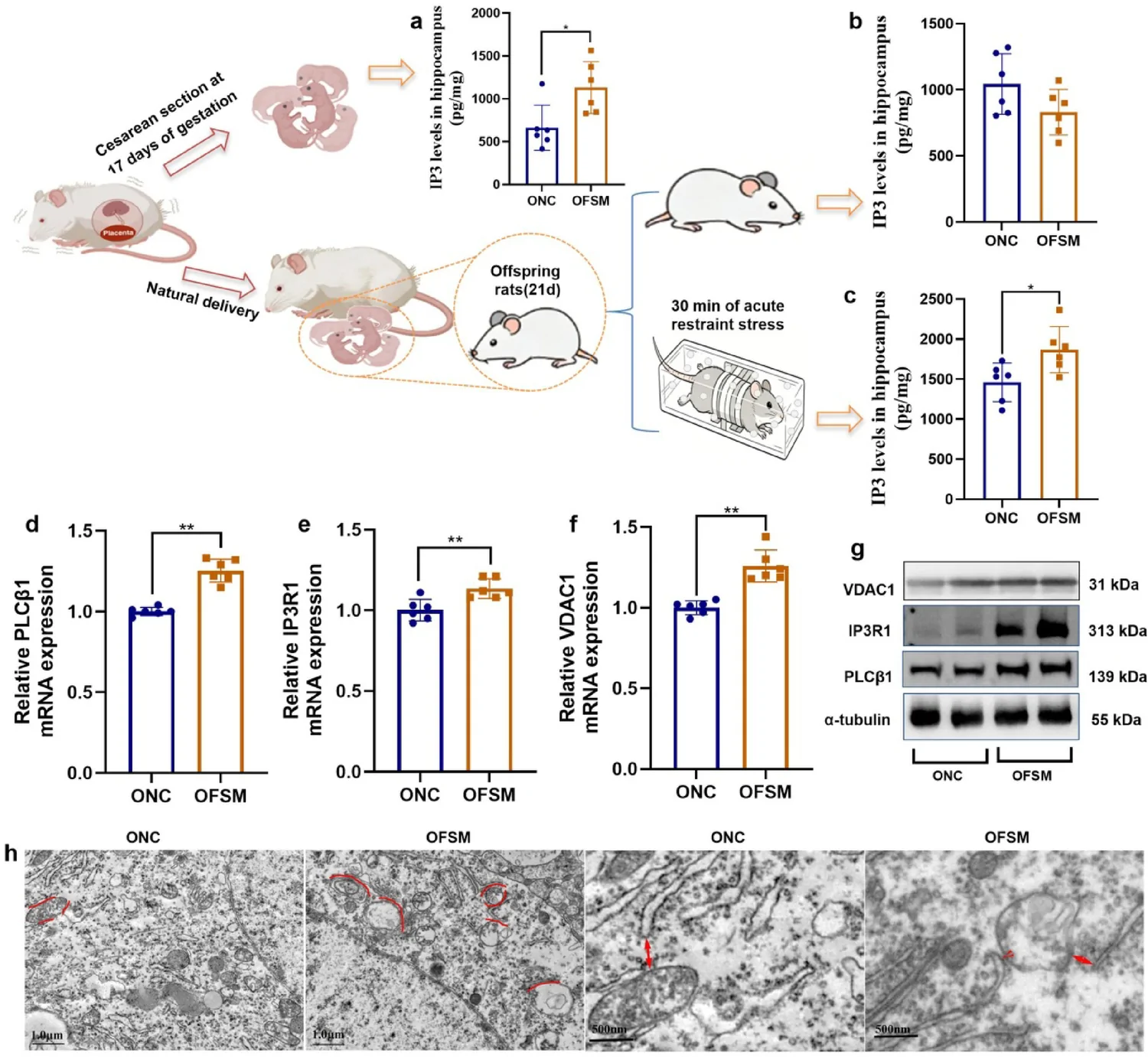
Effects of prenatal stress on hippocampal MAM structure and function in offspring. **a** The levels of hippocampal IP₃ in neonates. The levels of hippocampal IP₃ content at PND21 under basal conditions (**b**) and after acute restraint stress (**c**). **d-f** Relative mRNA expression of PLCβ1 and MAM-associated molecules (IP3R1 and VDAC1) at PND21 after acute stress; **g** representative images of western blots. **h** Representative transmission electron microscopy images of mitochondria-ER contacts in hippocampal neurons. Statistical significance was determined using Student’s t-test. *p < 0.05, **p < 0.01 vs. control group

OFSM neonates exhibited elevated hippocampal IP₃ at birth (Fig. 7a). At the early socialization stage, basal IP₃ levels did not differ between groups (Fig. 7b); however, 30-min acute restraint stress triggered a significant IP₃ increase in the OFSM group (Fig. 7c). Notably, sex‑stratified analyses revealed no significant differences between male and female offspring in any of these IP₃ measurements. Additionally, the OFSM group exhibited abnormally elevated PLCβ1 mRNA and protein expression, accompanied by upregulated MAM-associated mRNA and protein expression IP3R1 and VDAC1 (Fig. 7d and g). Ultrastructural analysis revealed enlarged MAM coverage and reduced mitochondria-ER distance in model pups (Fig. 7h).

**Fig. 8:**
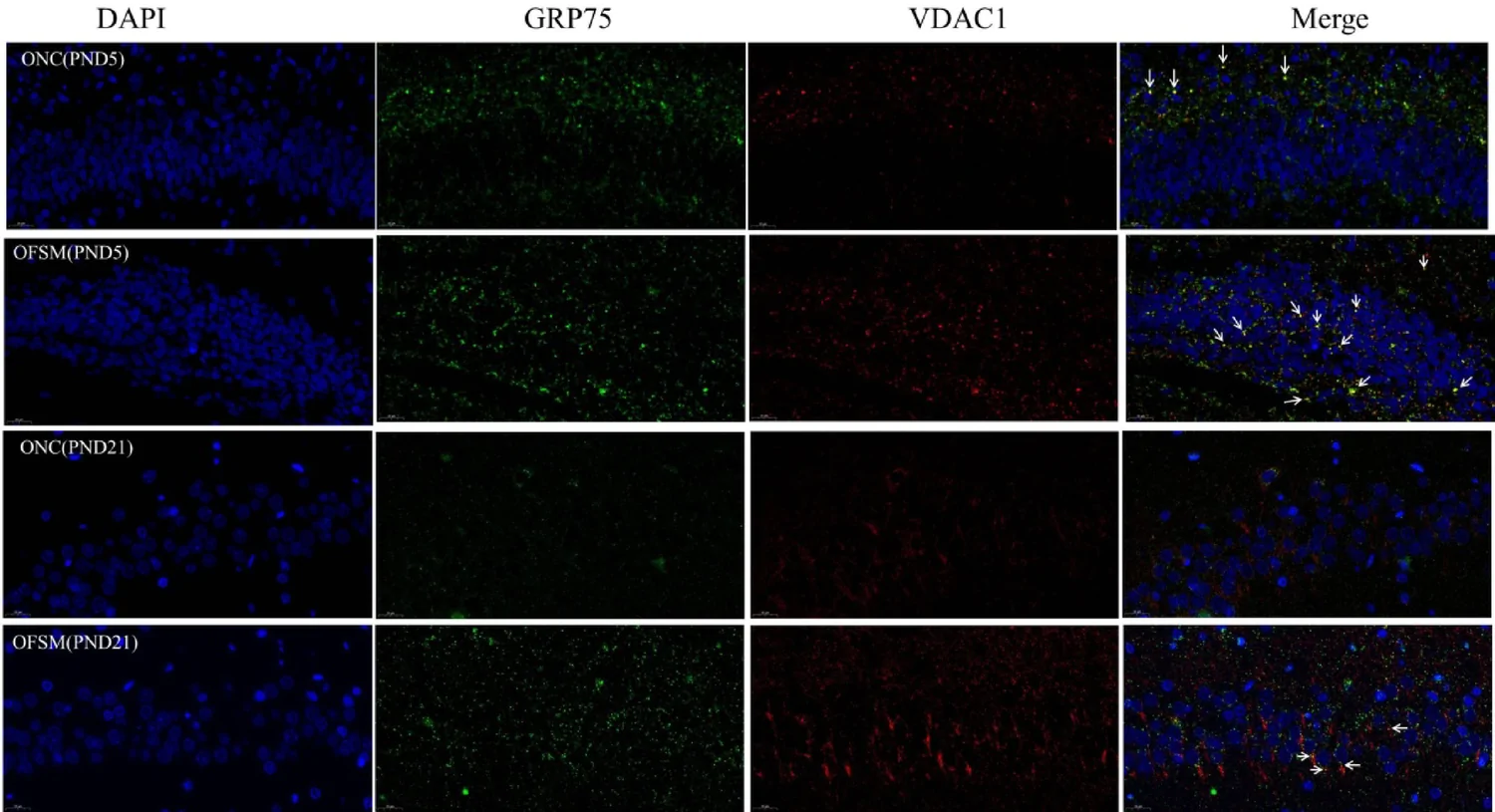
GRP75‑VDAC1 co‑localization in the offspring hippocampus. Immunofluorescence staining of GRP75 (green) and VDAC1 (red) in the offspring hippocampus at PND 5 and PND 21. Merged images show co‑localization (yellow), indicated by white arrows

To validate MAM remodeling at the molecular level, we performed GRP75‑VDAC1 immunofluorescence co‑localization (Fig. 8). In 5‑day‑old neonatal offspring, GRP75‑VDAC1 co‑localization was significantly increased in the OFSM group. At the early socialization stage, co‑localization remained elevated relative to controls but was significantly reduced compared with neonatal levels. These molecular findings are consistent with the ultrastructural evidence of MAM structural changes, together suggesting that MAM alterations occur at both the morphological and molecular levels.

### CRH Overexposure Upregulates the PLCβ1‑IP3R‑VDAC1 Pathway and Induces Mitochondrial Calcium Overload in SH‑SY5Y Cells, Reversed by CRHR1 Antagonism, PLCβ1 Knockdown, or MCU Inhibition

**Fig. 9:**
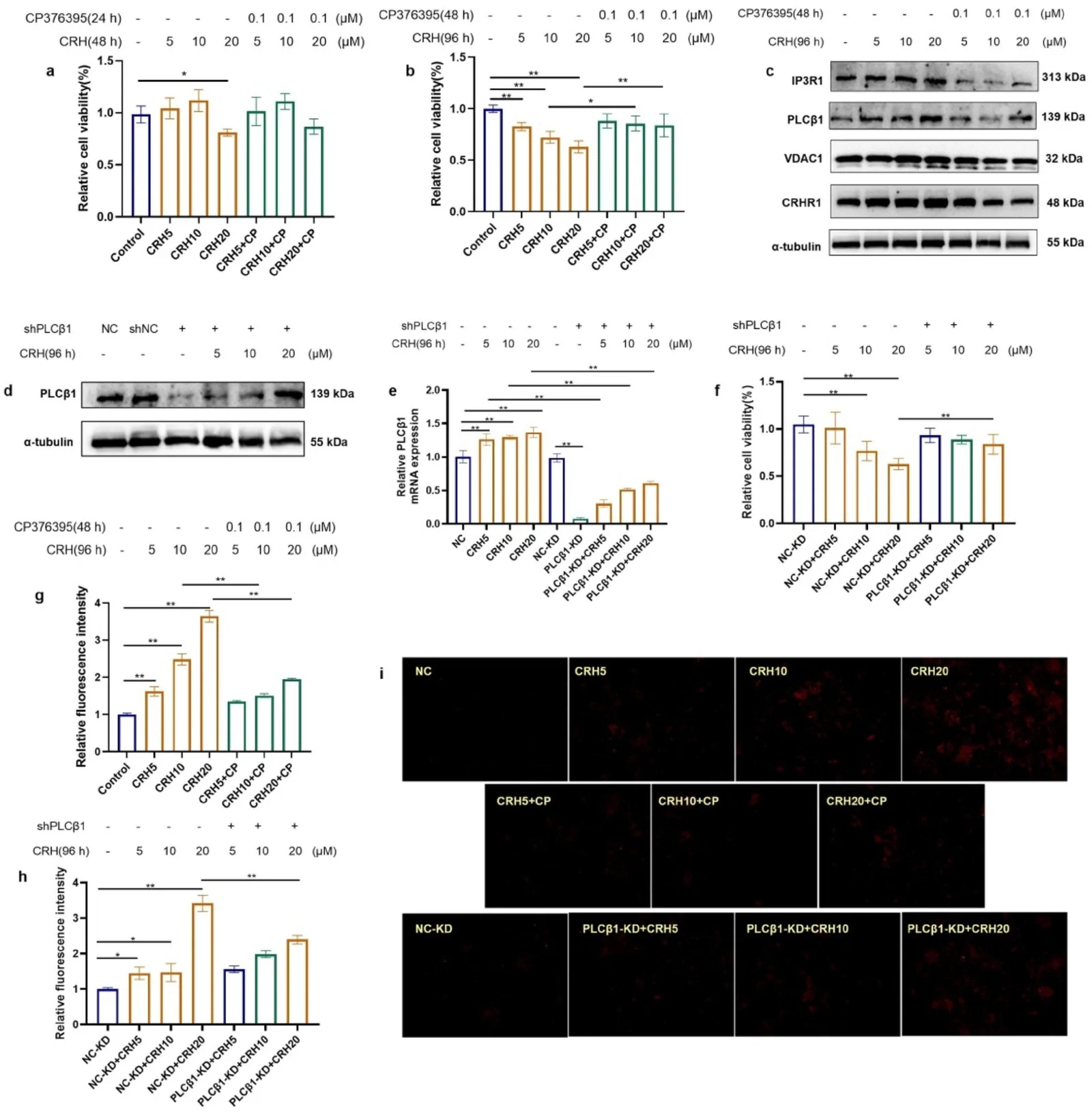
CRH induces mitochondrial Ca²⁺ overload and proliferation inhibition via the CRHR1-PLCβ1-VDAC1 axis in SH-SY5Y cells. Cell viability after 48 h (**a**) or 96 h (**b**) CRH treatment and the reversal effect of CRHR1 antagonist (CP376395) on CRH-induced proliferation inhibition. **c** Representative Western blot images of CRHR1, PLCβ1, IP3R1 and VDAC1 protein after 96 h CRH exposure, with or without CRHR1 antagonist. **d** Representative Western blot images of PLCβ1 protein in SH-SY5Y cells and PLCβ1-KD SH-SY5Y cells treated with indicated concentrations of CRH. **e** Relative PLCβ1 mRNA expression in SH-SY5Y cells and PLCβ1-KD SH-SY5Y cells treated with indicated concentrations of CRH. **f** Effect of stable PLCβ1 knockdown on CRH-induced proliferation inhibition. **g** Mitochondrial Ca²⁺ fluorescence intensity in SH-SY5Y cells after 96 h CRH treatment, with or without CRHR1 antagonist. **h** Mitochondrial Ca²⁺ levels after PLCβ1 knockdown and CRH treatment. **i** Representative images of mitochondrial Ca²⁺ labeled with Rhod-2 AM under different treatments, higher red fluorescence indicates elevated mitochondrial Ca²⁺ levels. Statistical significance was determined using one-way ANOVA followed by Tukey’s post hoc test for multiple comparisons. *p < 0.05, **p < 0.01

**Fig. 10:**
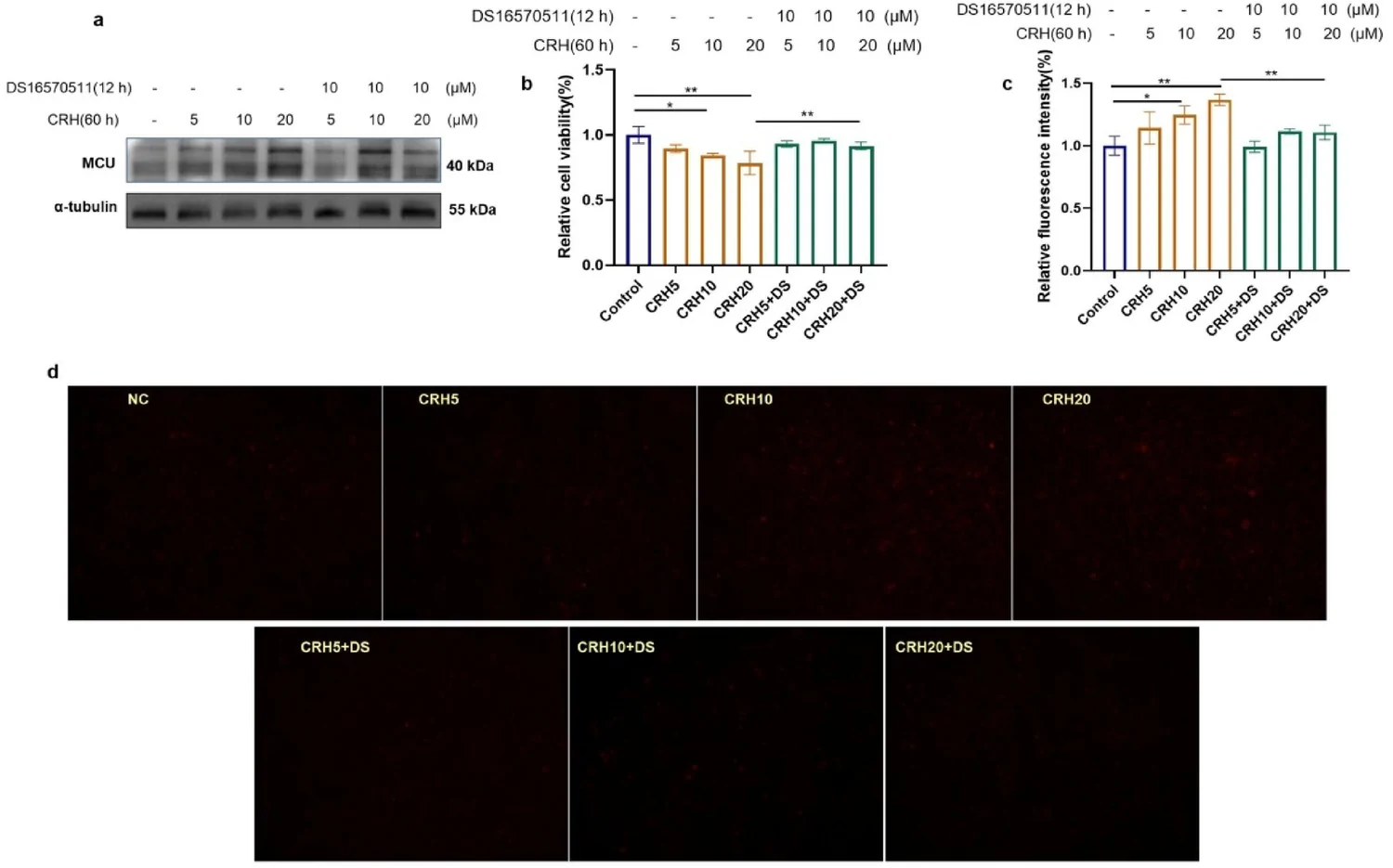
DS16570511 attenuates CRH‑induced proliferation inhibition and mitochondrial calcium overload in SH‑SY5Y cells. **a** Representative Western blot images of MCU protein after 60 h CRH exposure, with or without MCU antagonist (DS16570511). **b** Cell viability after 60 h CRH treatment and the reversal effect of MCU antagonist (DS16570511) on CRH-induced proliferation inhibition. **c** Mitochondrial Ca²⁺ fluorescence intensity in SH-SY5Y cells after 60 h CRH treatment, with or without MCU antagonist. **d** Representative images of mitochondrial Ca²⁺ labeled with Rhod-2 AM under different treatments, higher red fluorescence indicates elevated mitochondrial Ca²⁺ levels. Statistical significance was determined using one-way ANOVA followed by Tukey’s post hoc test for multiple comparisons. *p < 0.05, **p < 0.01

In SH‑SY5Y cells, CRH (20 µM for 48 h; 5–20 µM for 96 h) significantly inhibited proliferation, an effect rescued by the CRHR1 antagonist CP376395 (Fig. 9a and b). CRH (10–20 µM for 96 h) upregulated CRHR1, PLCβ1, and VDAC1 protein expression, an effect blocked by the antagonist CP376395 (Fig. 9c). In addition, mitochondrial Ca²⁺ increased after 5–20 µM CRH exposure for 96 h, prevented by CRHR1 inhibition (Fig. 9g and i). PLCβ1 knockdown (Fig. 9d, e) also alleviated CRH‑induced proliferation suppression (Fig. 9f) and reduced mitochondrial Ca²⁺ levels (Fig. 9h and i). Furthermore, DS16570511 (10 µM, 12 h) tended to reduce MCU expression induced by 20 µM CRH, significantly rescued both proliferation inhibition and mitochondrial Ca²⁺ elevation caused by 20 µM CRH, and restored the 10 µM CRH‑induced changes to control levels, though the latter effects were not statistically different from CRH alone (Fig. 10).

**Fig. 11:**
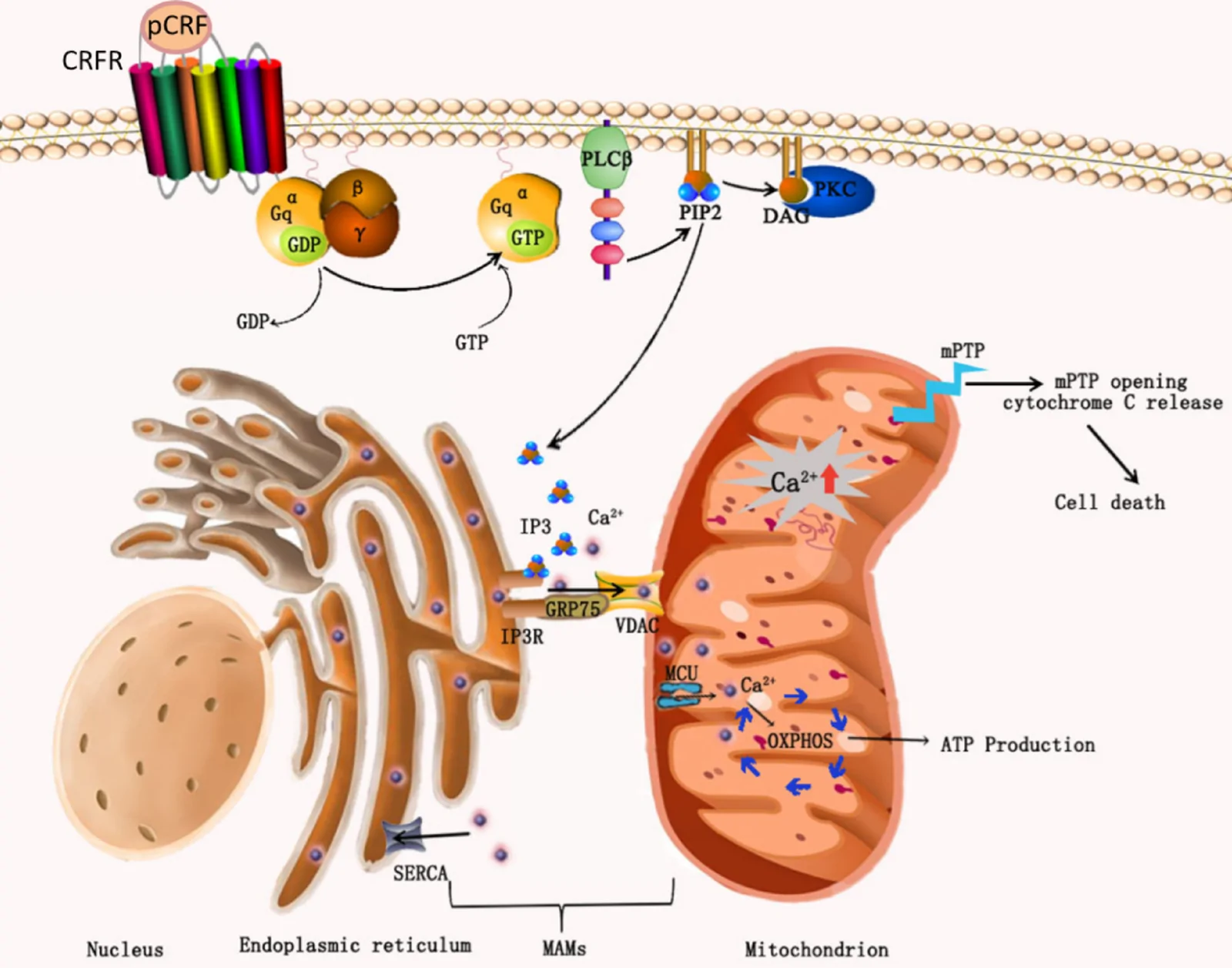
Regulation of the PLCβ1-IP3R-VDAC1 signaling pathway by the CRF-CRFR1 axis may mediate the association between prenatal fear stress and offspring hippocampal mitochondrial calcium overload and dysfunction

Our in vitro results demonstrate that CRH overexposure upregulates the PLCβ1‑IP3R‑VDAC1 pathway and induces mitochondrial calcium overload via CRHR1. Together with the in vivo observations of MAM structural remodeling and elevated IP3 levels in the offspring hippocampus, we speculate that CRH-CRHR1 signaling may influence the PLCβ1-IP3R-VDAC1 pathway and subsequent mitochondrial calcium overload, which may be involved in the MAM alterations in offspring hippocampus following prenatal stress (Fig. 11).

## Discussion

Mitochondrial dysfunction has emerged as a key mechanism linking early-life stress to adverse neurodevelopmental outcomes. Studies have demonstrated that prenatal stress can impair mitochondrial function in the offspring brain (Zhao et al. 2022). However, the specific molecular pathways through which prenatal fear stress disrupts offspring hippocampal mitochondrial function remain incompletely understood. To address this question, we performed a bioinformatic analysis to identify key genes and pathways potentially involved in prenatal stress-induced hippocampal mitochondrial damage. From the 34 overlapping genes related to mitochondrial damage, our bioinformatic analysis identified five differentially expressed genes enriched in neurodegenerative disease pathways: Ppp3r1, Psmd12, Psma4, Vdac1, and Plcb1. Among these, Ppp3r1, Psmd12, and Psma4 were downregulated, whereas Vdac1 and Plcb1 were upregulated. Further analysis revealed that PPP3R1, VDAC1, and PLCβ1 also belong to the calcium signaling pathway. This overlap suggested that calcium signaling pathway abnormalities may represent a critical link between prenatal stress and offspring hippocampal mitochondrial damage. Previous studies have independently linked PPP3R1, VDAC1, and PLCβ1 to neurodegenerative diseases. PPP3R1 serves as the regulatory subunit of calcineurin, and its low expression predicts Alzheimer’s disease onset (Zhou et al. 2021). VDAC1 is a key mitochondrial outer membrane channel regulating energy metabolism and apoptosis, and is overexpressed in Alzheimer’s disease brains (Cavallaro et al. 2025). PLCβ1 is the rate-limiting enzyme for IP3 generation, and its upregulation by Aβ42 induces neuronal calcium overload (Park et al. 2022). Notably, PLCβ1 and VDAC1 are core components of the PLCβ1-IP3R-VDAC1 calcium transfer axis, functionally mediating the direct transfer of calcium from the endoplasmic reticulum to mitochondria, whereas PPP3R1 primarily participates in downstream transcriptional regulation. Therefore, PLCβ1 and VDAC1 were selected as the primary targets for subsequent experimental validation. In addition, all the neurodegenerative disease‑related studies cited above focused on late‑adult or geriatric pathologies, whereas our bioinformatic predictions, together with subsequent in vivo data from prenatal fear stress pups, raise the possibility that abnormal activation of this pathway might be aberrantly activated during early postnatal development. This difference in the timing of disease onset suggests that prenatal fear stress may activate a calcium signaling pathway that is upregulated during aging. To test the functional relevance of the PLCβ1-IP3R-VDAC1 pathway identified by bioinformatics, we performed a series of in vivo experiments in a prenatal fear stress rat model. Specifically, we examined the expression of PLCβ1, IP3R and VDAC1, the level of IP3 as a functional readout of PLCβ1 activity, and the ultrastructural morphology of MAM in the offspring hippocampus. These experiments were designed to determine whether the pathway predicted by bioinformatics is indeed activated in vivo and whether it correlates with mitochondrial structural and functional alterations.

Previous studies have demonstrated that prenatal stress can program offspring HPA axis hyperreactivity through sustained maternal glucocorticoid exposure (Irwin et al. 2021). Consistent with the report, our model offspring exhibited latent HPA axis hyperresponsiveness, as evidenced by exaggerated CRF, ACTH, and corticosterone responses to acute restraint stress, along with upregulated hippocampal CRF receptor expression. These findings indicate that prenatal stress might affect the stress regulatory system in the offspring hippocampus, rendering it hyperreactive. This process might then represent an upstream event that contributes to alterations in offspring hippocampal mitochondrial function.

After confirming the stability of the model, we next sought to determine whether the PLCβ1-IP3R-VDAC1 calcium signaling axis predicted by bioinformatics is activated in the offspring hippocampus. PLCβ1 activation generates IP3 (Bill and Vines 2020; Chang et al. 2022), which binds to IP3R on MAM and triggers calcium release from the ER to mitochondria via the IP3R-GRP75-VDAC1 complex (Morris et al. 2021). Our observation of concurrent PLCβ1, IP3, IP3R1, and VDAC1 upregulation, along with increased GRP75‑VDAC1 co‑localization, indicates MAM remodeling and hyperactivation of calcium transfer in the offspring hippocampus, consistent with the bioinformatic prediction. PND 21 represents a critical developmental window in the rat hippocampus (Lauterborn et al. 2017). Given that hippocampal synaptic maturation and plasticity peak around this developmental stage, PND21 provides a window for detecting early molecular alterations that may precede or predict later cognitive deficits. The observation of PLCβ1-IP3R-VDAC1 pathway hyperactivation at this developmental stage raises the possibility that prenatal stress may influence mitochondrial calcium signaling during a period that is critical for hippocampal circuit formation.

In addition, in a complementary pilot experiment, we examined the effects of dexamethasone injection, which mimics downstream glucocorticoid signaling of prenatal stress, and observed similar alterations in offspring hippocampal protein expression as seen in the fear stress model. However, given that dexamethasone was administered via subcutaneous injection, which introduces an additional stressor not present in the passive fear stress model, these data were not included in the main text but are available in the Supplementary Materials 1. Furthermore, GRP75‑VDAC1 co‑localization signal declined from birth to early socialization, indicating dynamic regulation of MAM contacts during postnatal development, though its functional significance remains to be clarified. These results are similar to previous reports, suggesting that hyperactivation of PLCβ1-IP3R-VDAC1 signaling pathway may be associated with developmental neurotoxicity. Gao et al. demonstrated that maternal cyfluthrin exposure during pregnancy upregulates the IP3R-GRP75-VDAC1 pathway in offspring hippocampus, leading to neuronal apoptosis and synaptic plasticity impairments (Gao et al. 2024). Our findings indicate that the PLCβ1-IP3R-VDAC1 signaling pathway may be involved in the effects of prenatal stress on offspring hippocampal mitochondrial function.

In addition, behavioral assessments revealed significant sex differences in offspring following prenatal fear stress. Male pups exhibited more pronounced anhedonia and irritability, whereas female pups showed longer tail suspension immobility; both sexes displayed reduced exploratory behavior and spatial memory deficits, although the temporal patterns of escape latency impairment differed between sexes. These findings are consistent with previous reports that prenatal stress exerts sex-dependent behavioral effects, with males generally showing greater vulnerability (Van den Hove et al. 2013; Schulz et al. 2011; Gaetano et al. 2025). Behavioral results suggested that sex‑balanced sampling should be considered in mechanistic analyses. Due to the uneven sex distribution of offspring (with males comprising the vast majority), we ensured balanced detection of both sexes for quantitatively reliable measures, including HPA axis‑related parameters and hippocampal IP3 levels. However, for protein and RNA expression data, the limited number of female offspring precluded sex‑stratified statistical analysis; therefore, data from both sexes were combined for analysis. Given that this combined-sex analysis served only as an initial screening for core pathways shared between sexes, its influence on pathway identification is minimal. Moreover, this initial screening strategy primarily references a 2025 transcriptomic study demonstrating that mitochondrial dysfunction is a central mechanism shared by both sexes following prenatal stress (Di Benedetto et al. 2025). Earlier work also showed that prenatal stress attenuates normal gender differences in hippocampal gene expression, reducing sex-dependent differences from thousands of genes to only 191, while simultaneously inducing shared transcriptional changes in both sexes (Biala et al. 2011). Moreover, sex-stratified analyses of IP₃ assays revealed no significant differences between males and females, further supporting the validity of pooling data for mechanistic readouts where sex differences were not the primary focus. Nonetheless, we acknowledge that the use of pooled samples carries certain limitations. Future studies with larger sample sizes and sex-stratified analyses are warranted to elucidate how sex‑specific molecular modulations contribute to the distinct behavioral phenotypes reported here.

Our in vivo data showed that prenatal fear stress induced maternal HPA axis hyperactivation and elevated CRF levels in both the placenta and neonatal brain, and concomitantly hyperactivated the PLCβ1‑IP3R‑VDAC1 pathway. However, whether there is a direct link between elevated CRF signaling and pathway activation remains unclear. Given that excessive CRF exposure during development has been reported to interfere with fetal neurogenesis (Kassotaki et al. 2021), we considered the possibility that the CRF‑CRFR1 signaling might regulate the bioinformatically predicted PLCβ1‑IP3R‑VDAC1 pathway. To explore this, we exposed SH‑SY5Y human neuroblastoma cells to CRH and monitored changes in cell viability, pathway component levels, and mitochondrial calcium. In vitro, excessive CRH exposure activated the PLCβ1‑IP3R‑VDAC1 pathway, resulting in mitochondrial calcium overload and proliferation inhibition, as evidenced by rescue with CRHR1 antagonism, PLCβ1 knockdown, or MCU inhibition. These observations raise the possibility that excessive CRH exposure inhibits SH‑SY5Y cell proliferation, and that this effect is associated with mitochondrial calcium overload resulting from hyperactivation of the PLCβ1‑IP3R‑VDAC1 pathway. These findings are consistent with the in vivo data and bioinformatic predictions.

CRHR1 is a class B G protein‑coupled receptor that classically signals via the Gs‑cAMP‑PKA pathway (Yang and Geng 2023). Traditionally class B GPCRs are not considered activators of PLCβ. However, emerging evidence indicates that CRHR exhibits signaling promiscuity and can activate PLCβ through Gq protein coupling in specific cellular contexts or under stress conditions (Grammatopoulos 2012; Dautzenberg et al. 2004). Dautzenberg et al. first demonstrated that activation of CRF1 and CRF2 (a) receptors in HEK293 cells elicits PLC‑dependent IP3 accumulation and calcium mobilization, which was completely blocked by the PLC‑specific inhibitor U‑73,122 (Dautzenberg et al. 2004). Sheng et al. observed that CRH induces PLC‑β3 phosphorylation in primary hippocampal neurons, and blocking PLC- but not PKA- abolished the CRH‑induced depression of NMDA currents (Sheng et al. 2008). Gutknecht et al. further showed that Gq/11 proteins play a key role in this signaling (Gutknecht et al. 2009). Collectively, these findings demonstrate that CRHR1 can indeed signal through the non‑canonical Gq‑PLCβ pathway in specific cellular backgrounds. These reports further support our speculation that excessive CRH exposure may cause neuronal injury, an effect that may be associated with mitochondrial calcium overload resulting from hyperactivation of the PLCβ1‑IP3R‑VDAC1 pathway. However, we acknowledge that SH-SY5Y cells, as a neuroblastoma cell line, may not fully recapitulate the complexity of the in vivo hippocampal environment. Future validation in primary hippocampal neurons or in vivo models will be required to confirm the physiological relevance of these findings.

Notably, recent studies have revealed that CRH-CRHR1 activation might impact mitochondrial function through mechanisms distinct from calcium transfer. Battaglia et al. reported that in hippocampal neurons, stress-induced CRHR1 activation appears to promote mitochondrial fission via the NF-kB-DRP1 axis, which was associated with bioenergetic defects and reduced neuronal activity, with these effects being reversible (Battaglia et al. 2020). Zhang et al. further showed that CRHR1 upregulation may damage mitochondria via the cAMP/Ras/MAPK pathway in colonocytes, suggesting that the CRH-CRHR1-mitochondria axis might mediate communication between the brain and peripheral systems (Zhang et al. 2024). Together, these findings indicate that CRH-CRHR1 signaling regulates mitochondrial function through multiple mechanisms, potentially in a tissue-specific manner. Our study extends this emerging picture by raising the possibility of the PLCβ1-IP3R-VDAC1 pathway may be an additional effector pathway, which appears in conjunction with CRH-CRHR1 signaling and correlates with mitochondrial calcium overload. Distinct from the previously reported fission-related and MAPK-mediated pathways, this regulatory node operates at the level of mitochondrial calcium transfer, suggesting that CRH-driven mitochondrial dysfunction may involve multiple parallel molecular routes. Whether and how these pathways interact in the developing hippocampus remains to be further investigated.

Collectively, this study preliminarily explored the potential mechanisms underlying prenatal fear stress‑induced hippocampal mitochondrial damage in offspring by integrating bioinformatics, in vivo, and in vitro approaches. Our results suggest that the CRH-CRHR1 axis may influence the PLCβ1-IP3R-VDAC1 pathway, accompanied by MAM remodeling and mitochondrial calcium overload, thereby potentially contributing to prenatal stress-induced hippocampal mitochondrial damage in offspring. Furthermore, it should be acknowledged that our bioinformatics analysis is predictive in nature, providing correlative clues rather than causal evidence. The subsequent PLCβ1 knockdown and CP376395 antagonism experiments have provided preliminary functional validation of this pathway, but definitive causal establishment will require neuron‑specific CRHR1 knockout animal models and mitochondria‑associated endoplasmic reticulum membrane‑targeted intervention studies.

## Supplementary Information

Below is the link to the electronic supplementary material.

Supplementary Material 1

Supplementary Material 2

Supplementary Material 3

Supplementary Material 4

## Data Availability

No datasets were generated or analysed during the current study.

## Abbreviations

CNS: Central nervous system
ANS: Autonomic nervous system
CRH: Corticotropin-releasing hormone (also termed CRF in rodents)
PLCβ1: Phospholipase C beta 1
GPCR: G protein-coupled receptor
MAM: Mitochondrial-associated endoplasmic reticulum membrane
IP3R: Inositol triphosphate receptor
GRP75: Glucose regulated protein 75
VDAC: Voltage dependent anion channel
HPA: Hypothalamic-pituitary-adrenal
NC: Normal control
FSM: Fear stress model
SPT: Sucrose preference test
TST: Tail suspension test
ACTH: Adrenocorticotropic hormone
CORT: Corticosterone
ELISA: Enzyme-linked immunosorbent assay
WB: Western blot
qPCR: Quantitative PCR
PND: Postnatal days
MWM: Morris water maze
OFT: Open field test
TEM: Transmission electron microscopy
HPG: Hypothalamic-pituitary-gonadal axis
NPC: Neural progenitor cell
ASD: Autism spectrum disorder
IP3: Inositol 1,4,5-Trisphosphate
