# Supplementary material for: The CRF‑CRFR1 Axis Mediates Prenatal Fear Stress‑Induced Hippocampal Mitochondrial Damage in Offspring via MAM Remodeling and Calcium Overload
Source: Cell Mol Neurobiol. 2026 Jul 19;46:151. doi: 10.1007/s10571-026-01780-0 (PMC13661846; doi:10.1007/s10571-026-01780-0)
Supplement: Supplementary file 1 [file 10571_2026_1780_MOESM1_ESM.pdf]

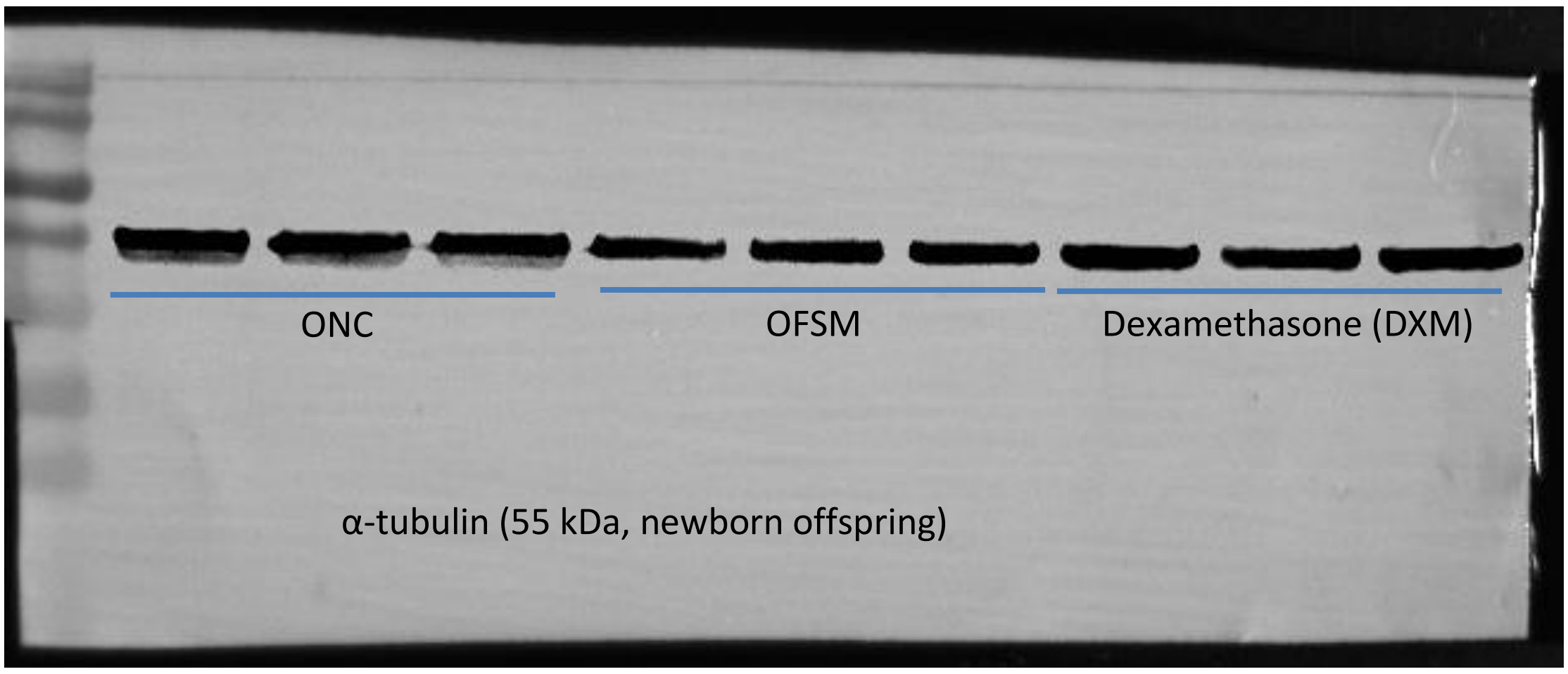
ONC
OFSM
Dexamethasone (DXM)
α-tubulin (55 kDa, newborn offspring)

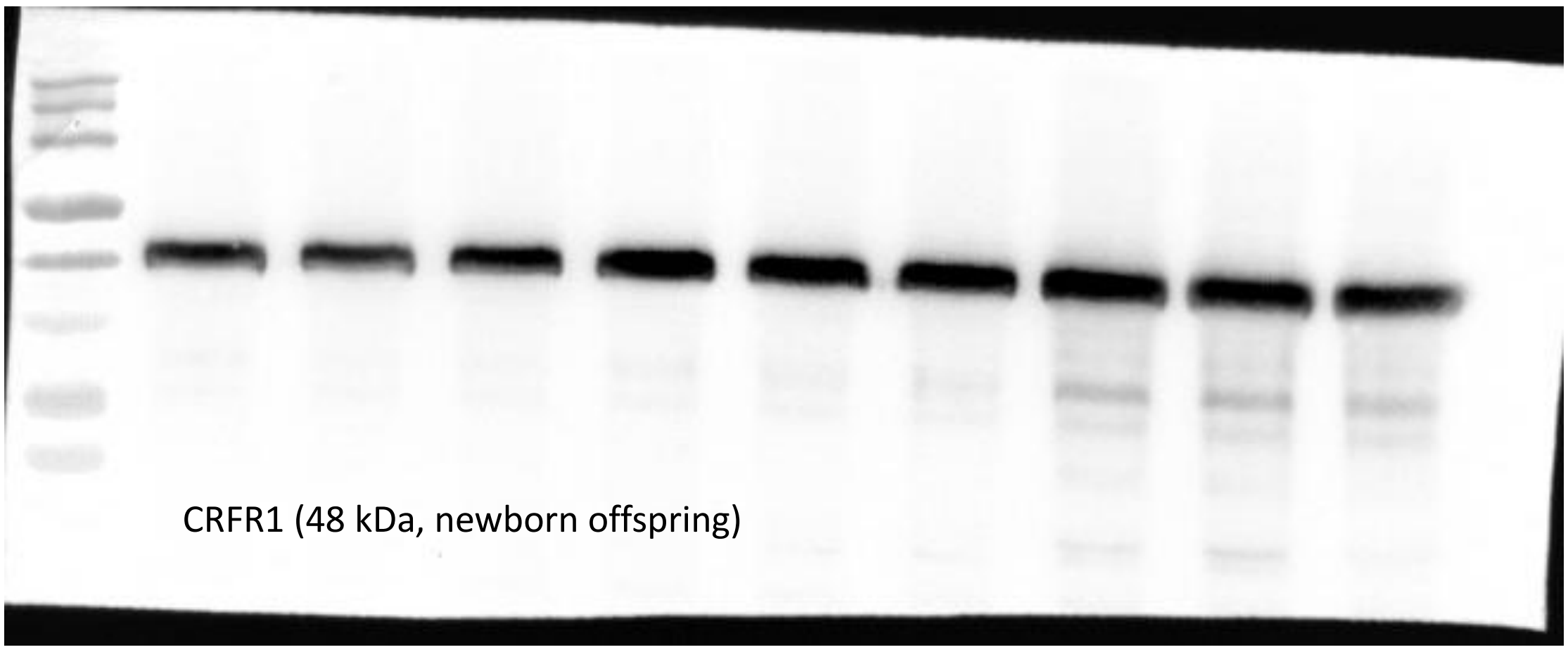
CRFR1 (48 kDa, newborn offspring)

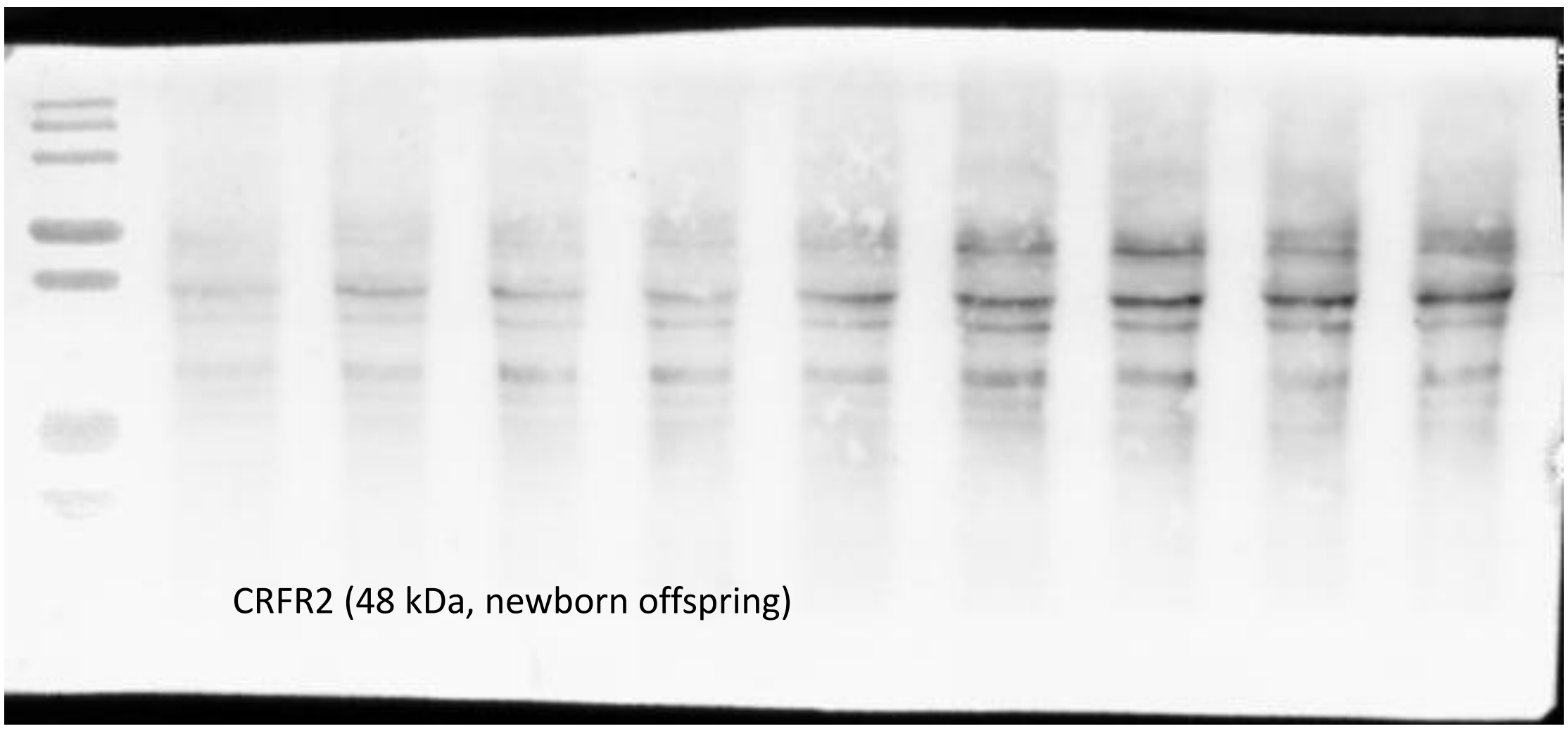
CRFR2 (48 kDa, newborn offspring)

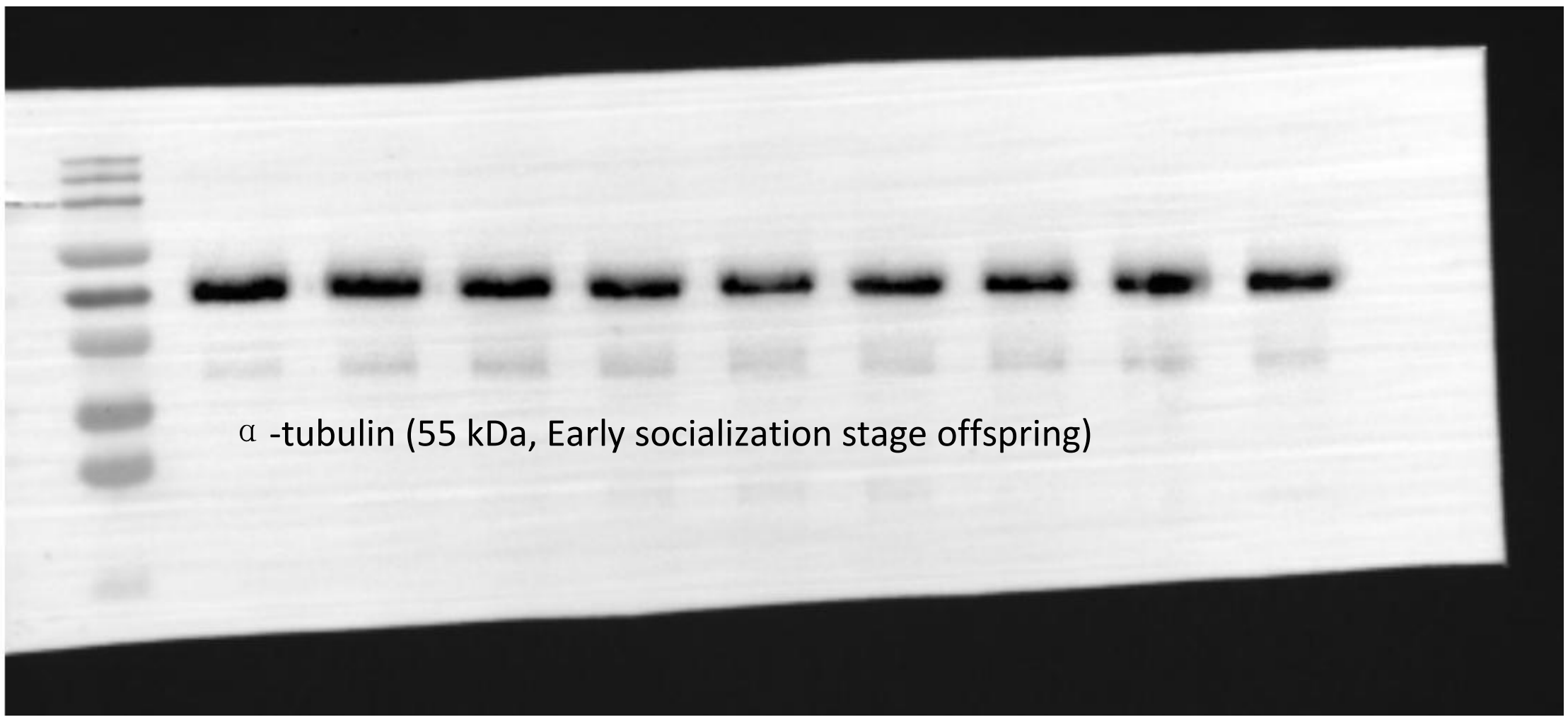
α -tubulin (55 kDa, Early socialization stage offspring)

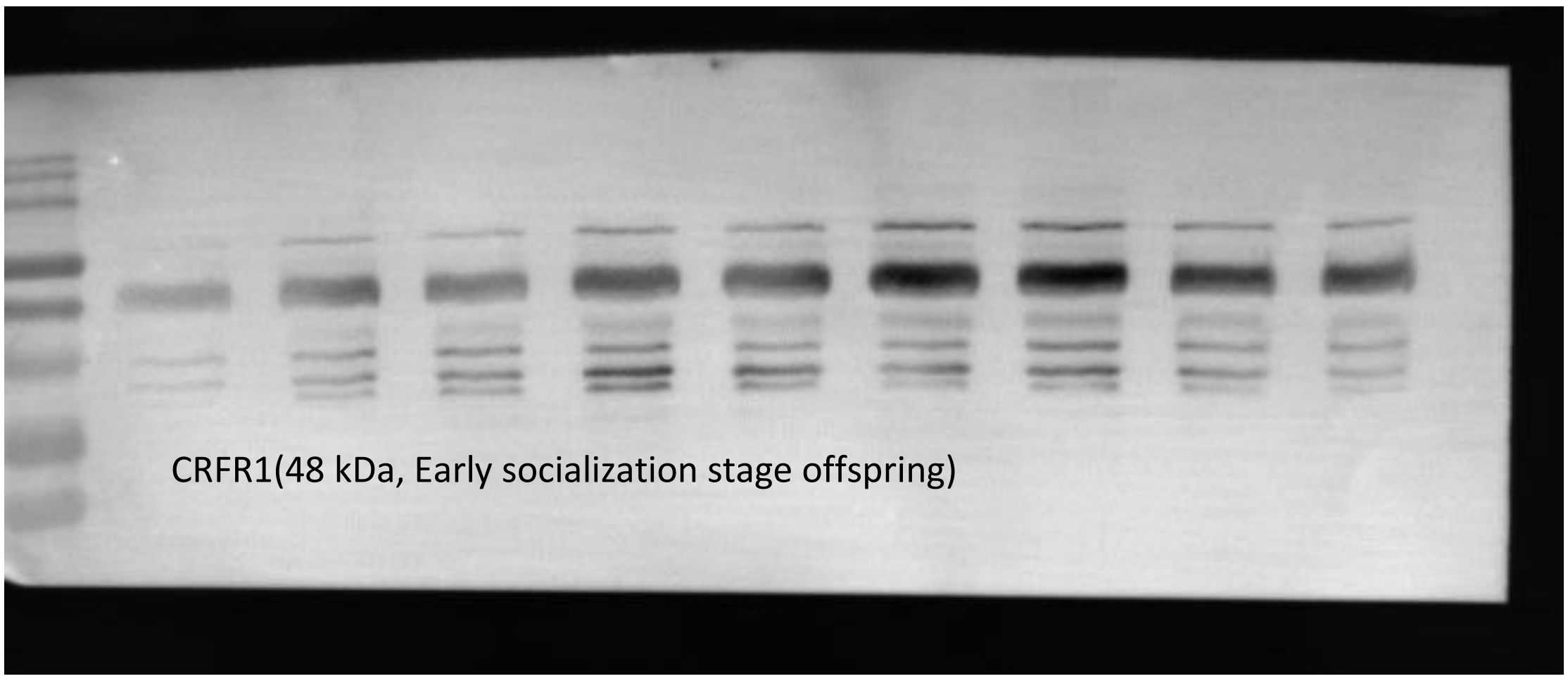
CRFR1(48 kDa, Early socialization stage offspring)

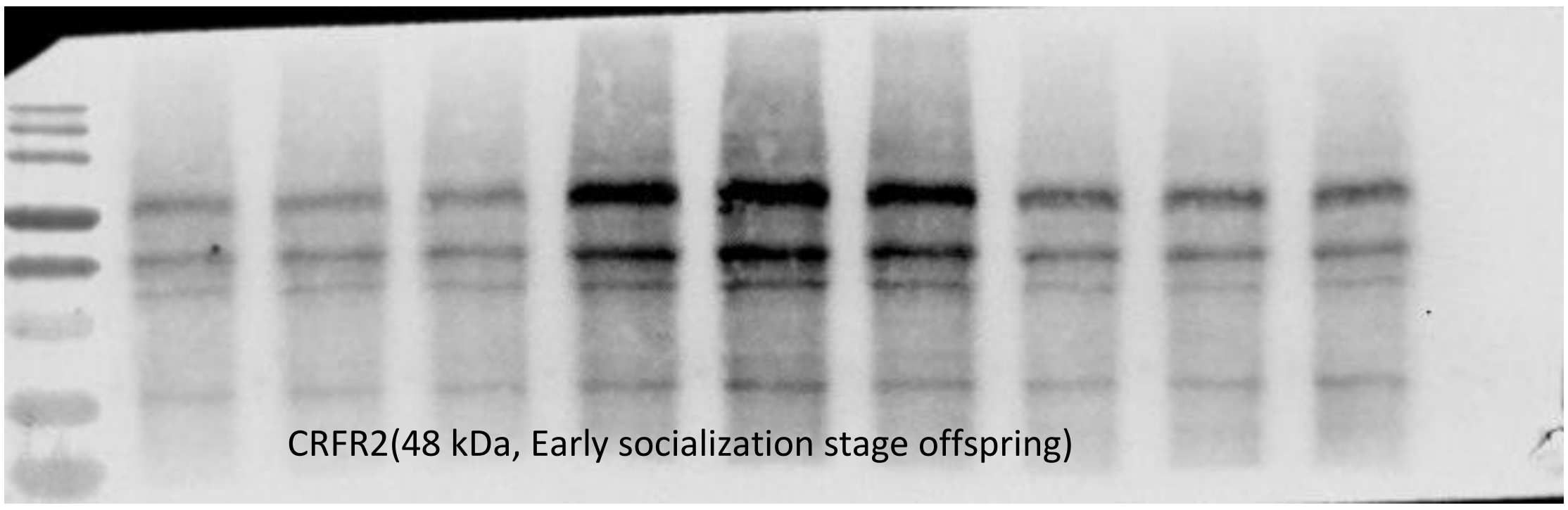
CRFR2(48 kDa, Early socialization stage offspring)

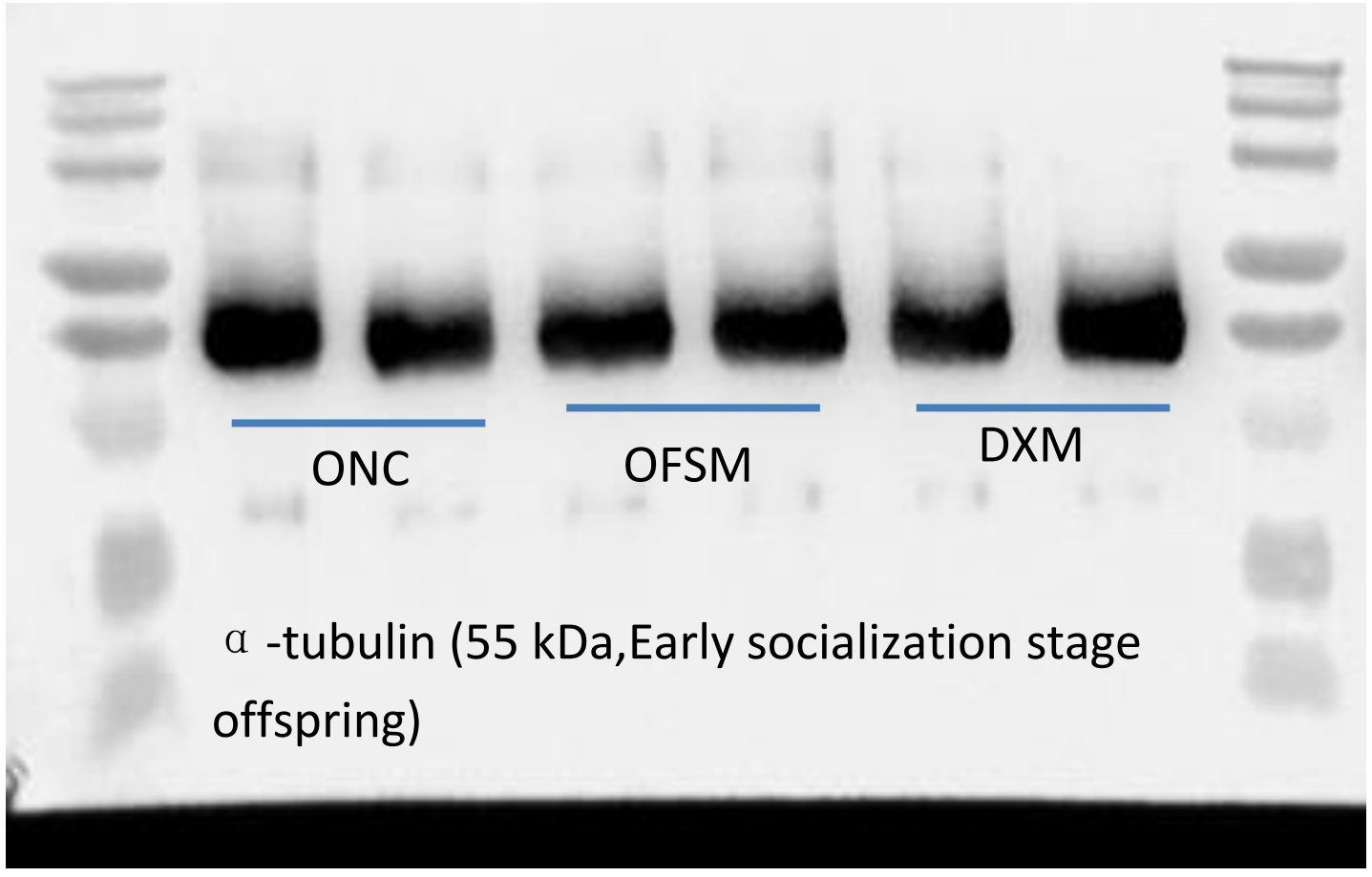
ONC
OFSM
DXM
α -tubulin (55 kDa,Early socialization stage offspring)

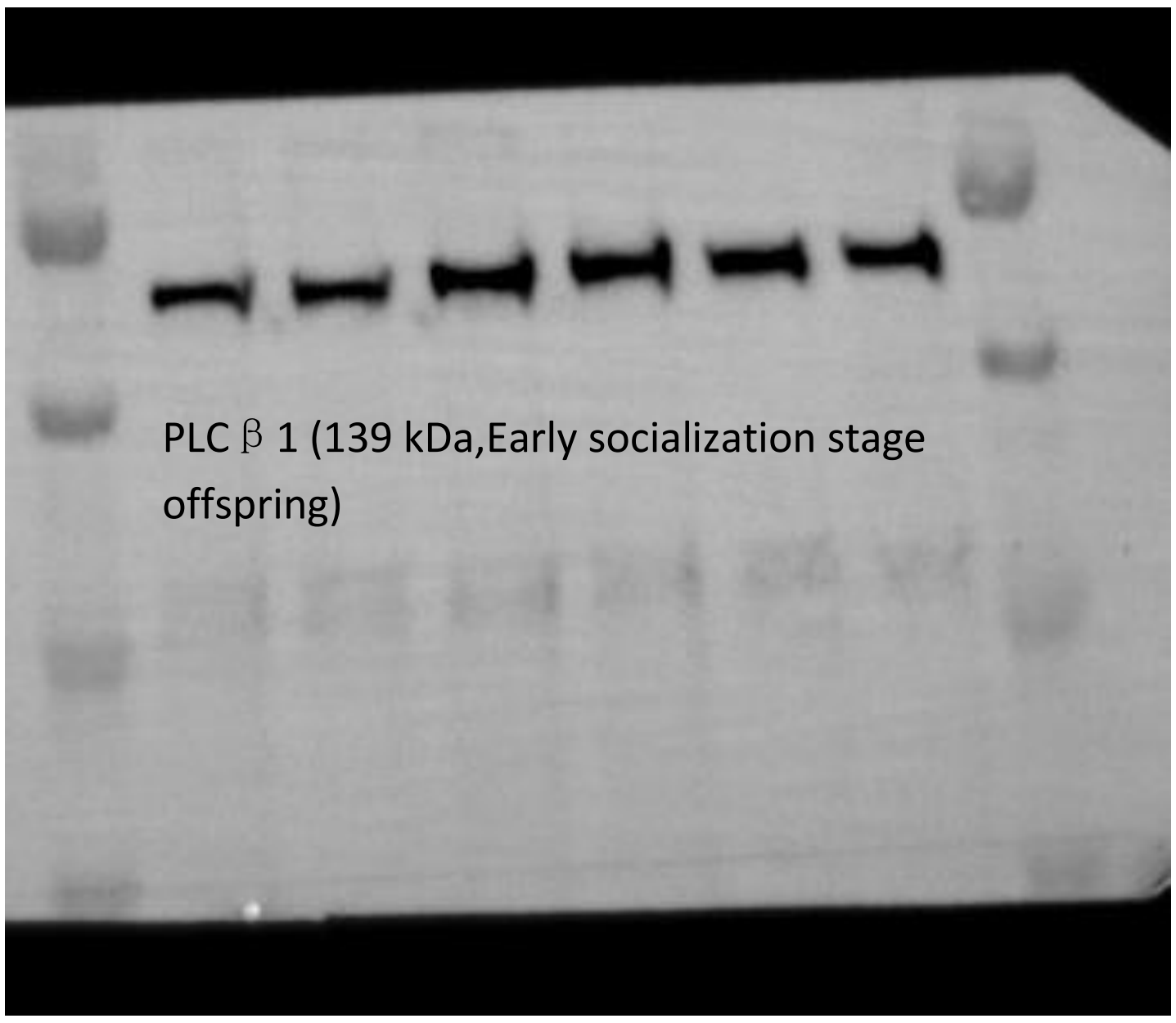
PLC β 1 (139 kDa,Early socialization stage offspring)

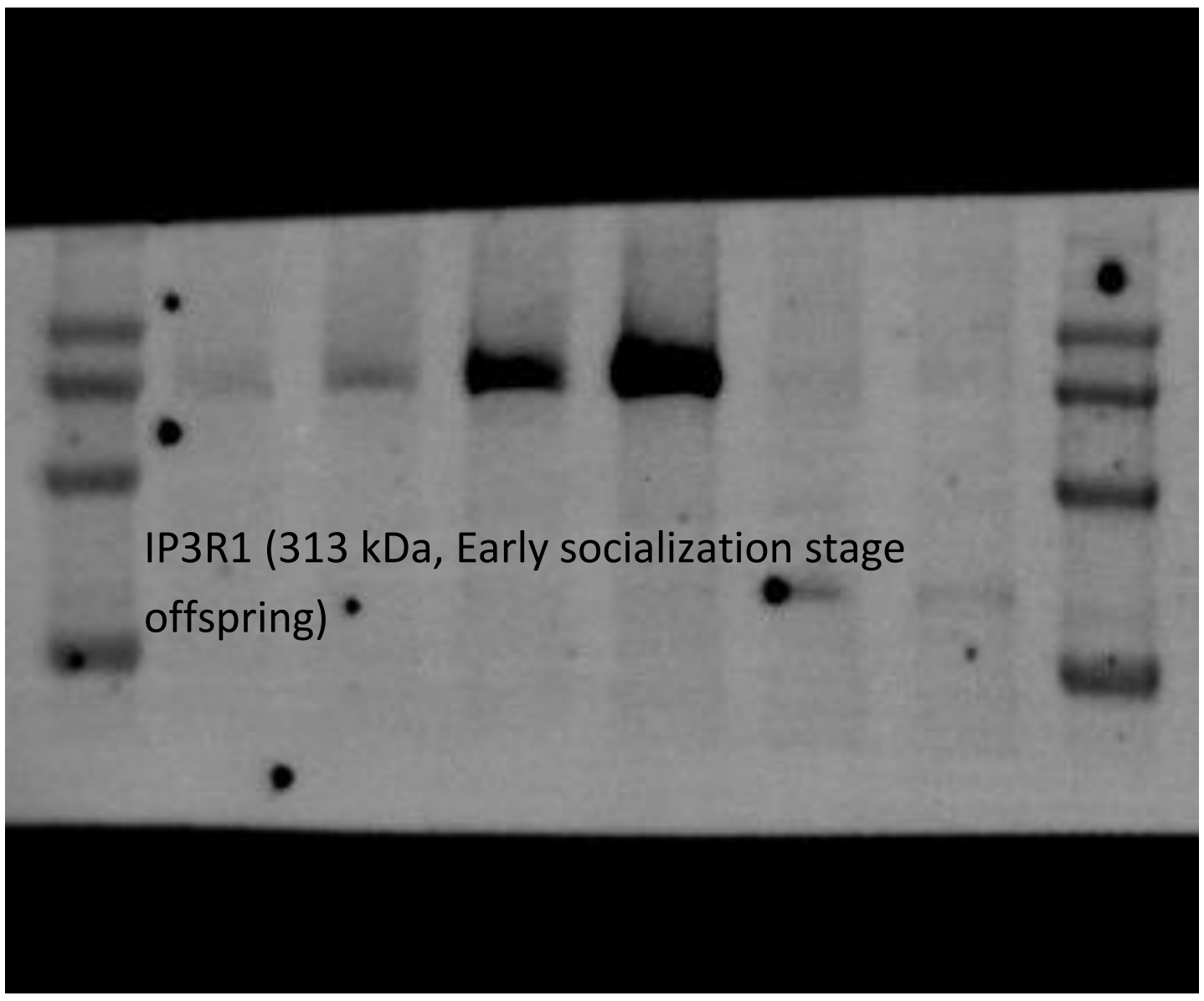
IP3R1 (313 kDa, Early socialization stage offspring)

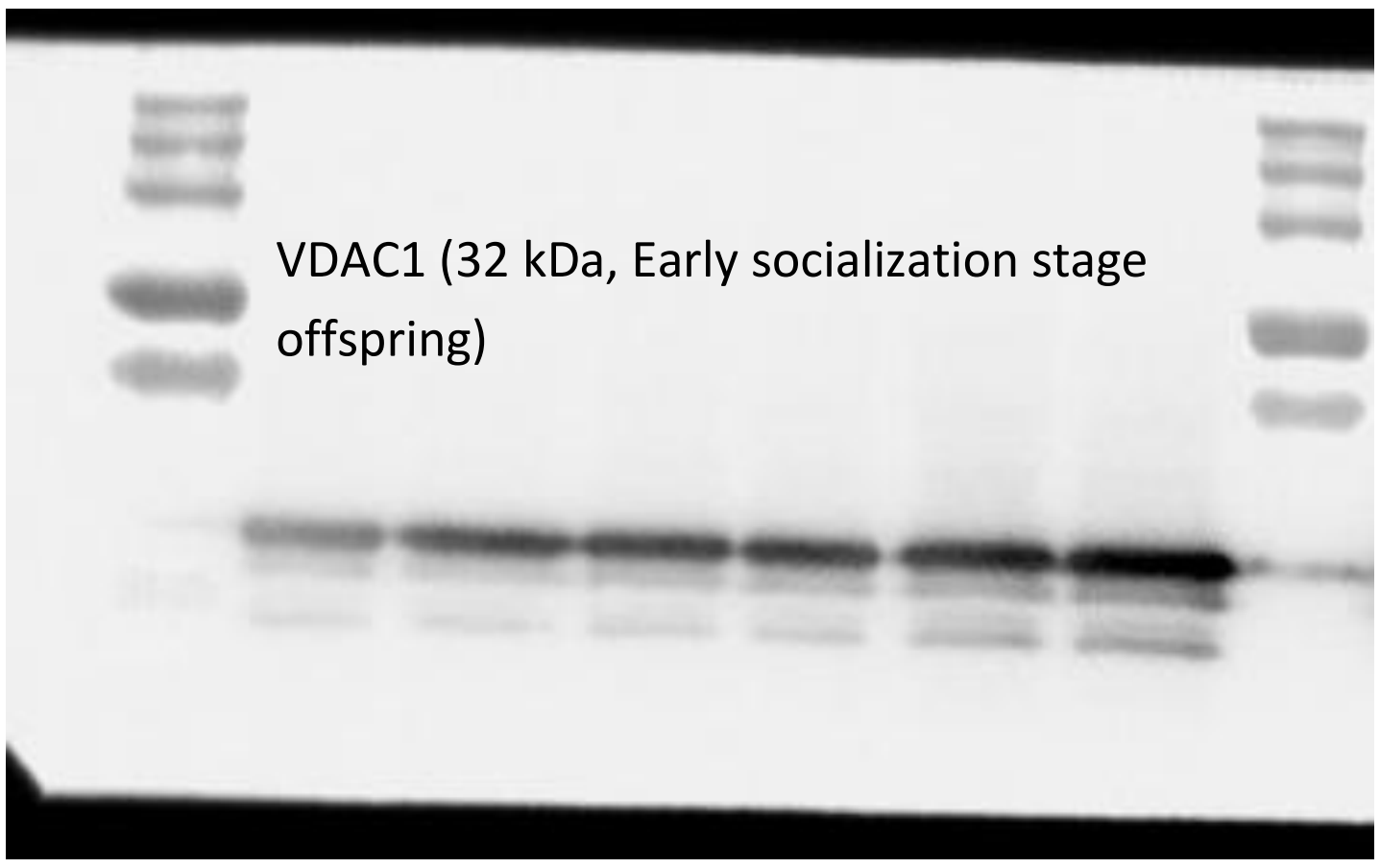
VDAC1 (32 kDa, Early socialization stage offspring)

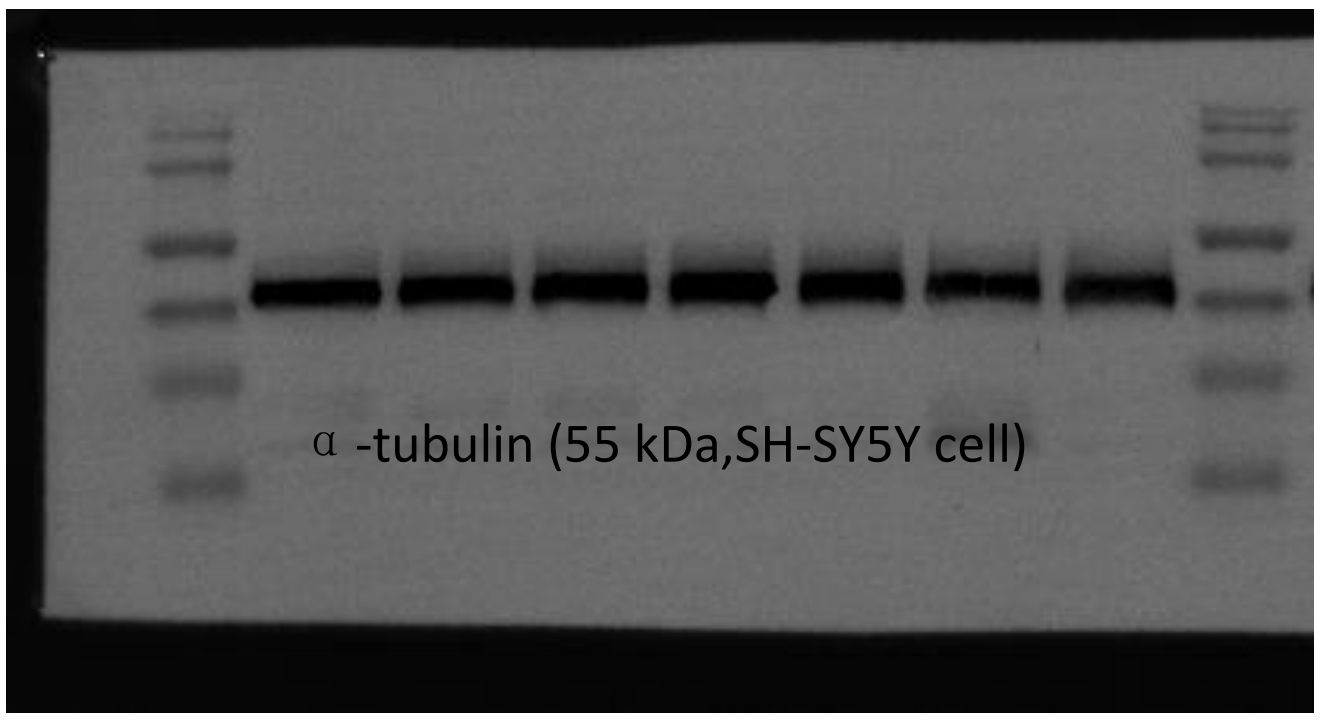
α -tubulin (55 kDa,SH-SY5Y cell)

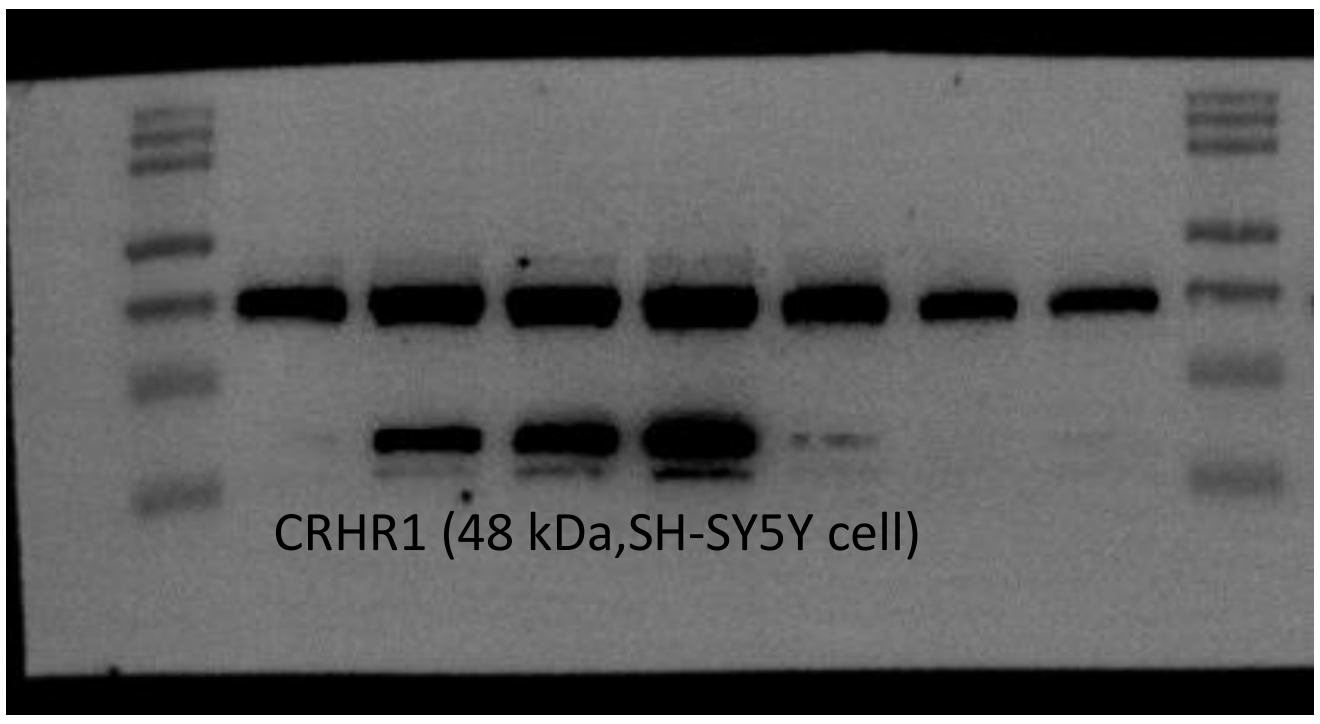
CRHR1 (48 kDa,SH-SY5Y cell)

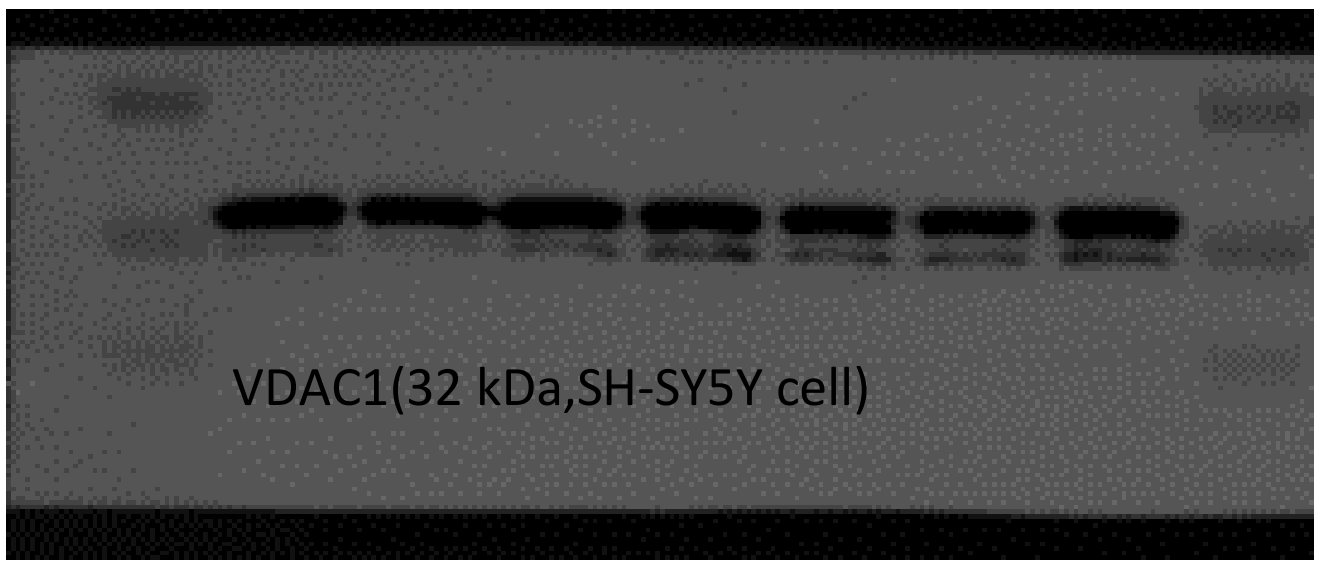
VDAC1(32 kDa,SH-SY5Y cell)

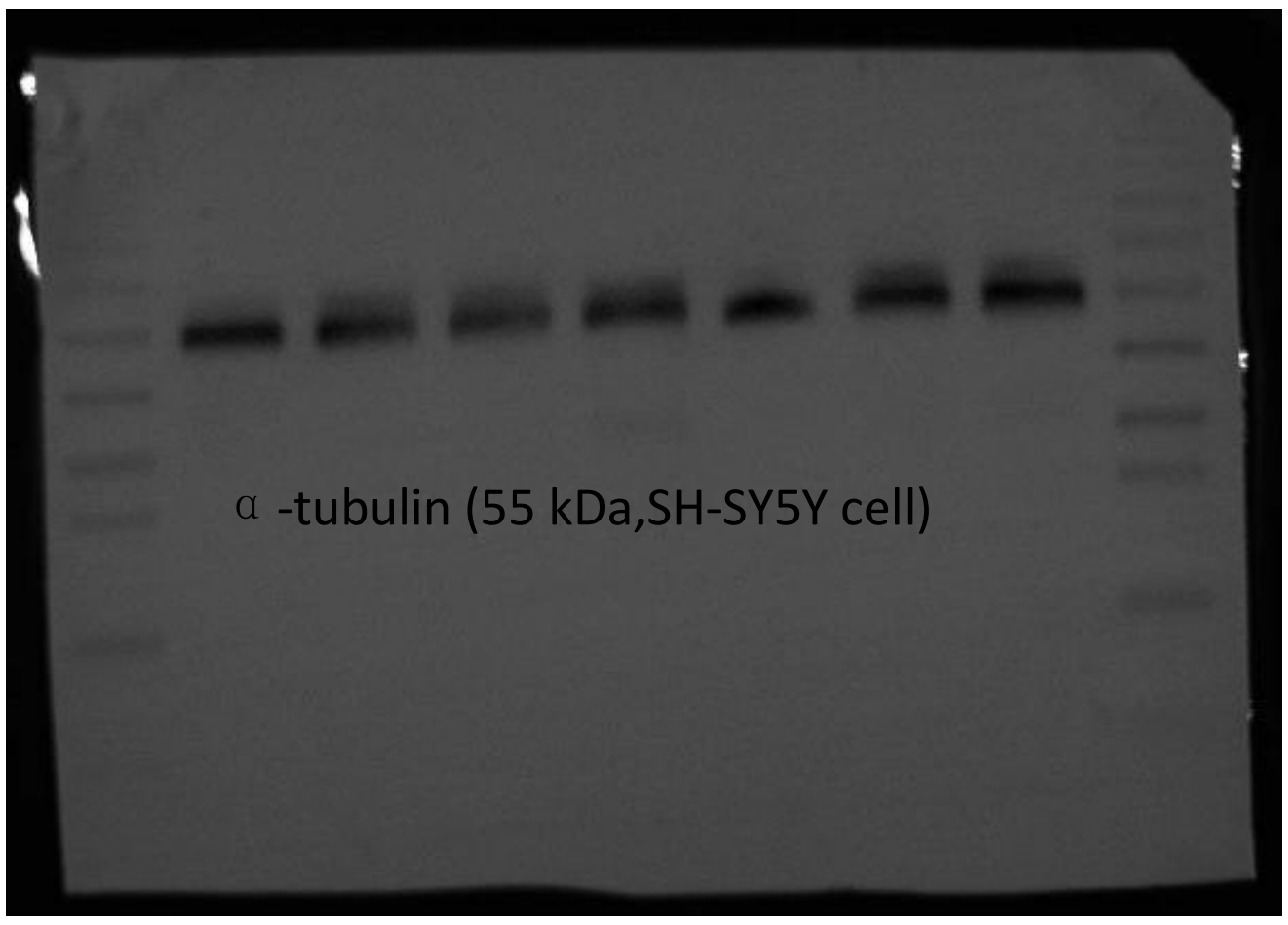
α -tubulin (55 kDa,SH-SY5Y cell)

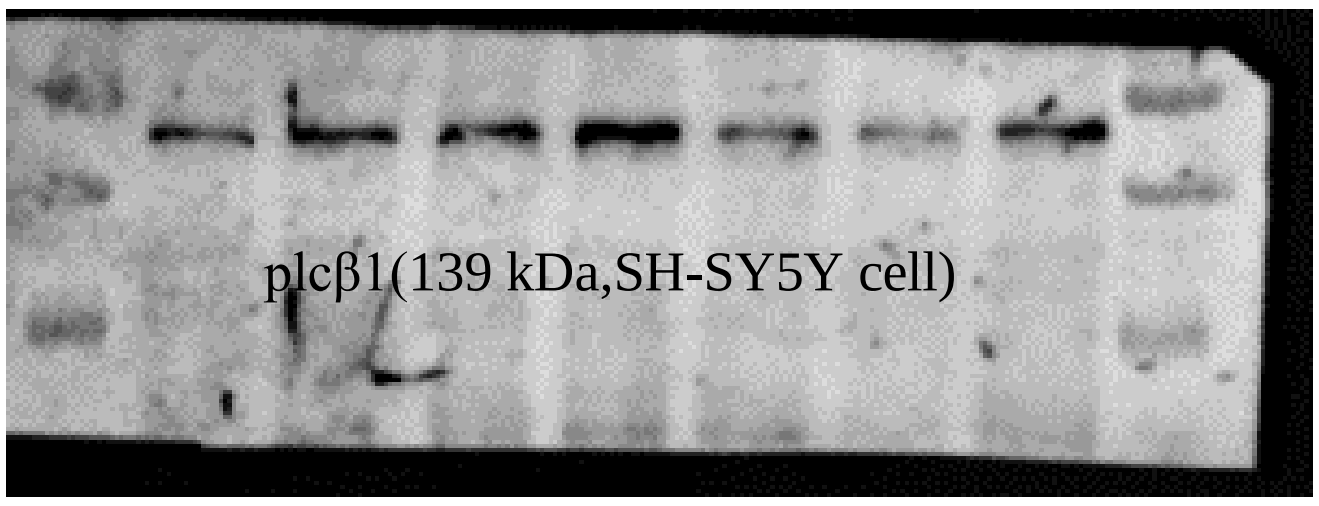
plcβ1(139 kDa,SH-SY5Y cell)

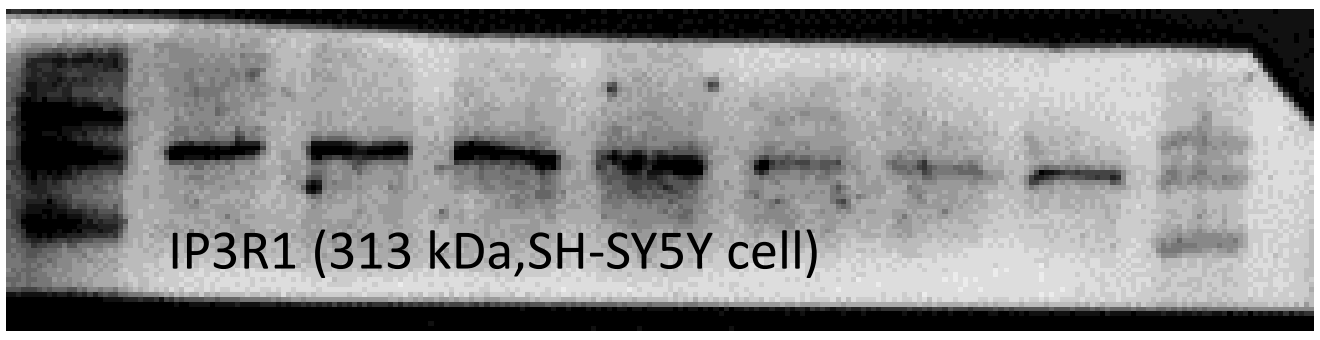
IP3R1 (313 kDa,SH-SY5Y cell)

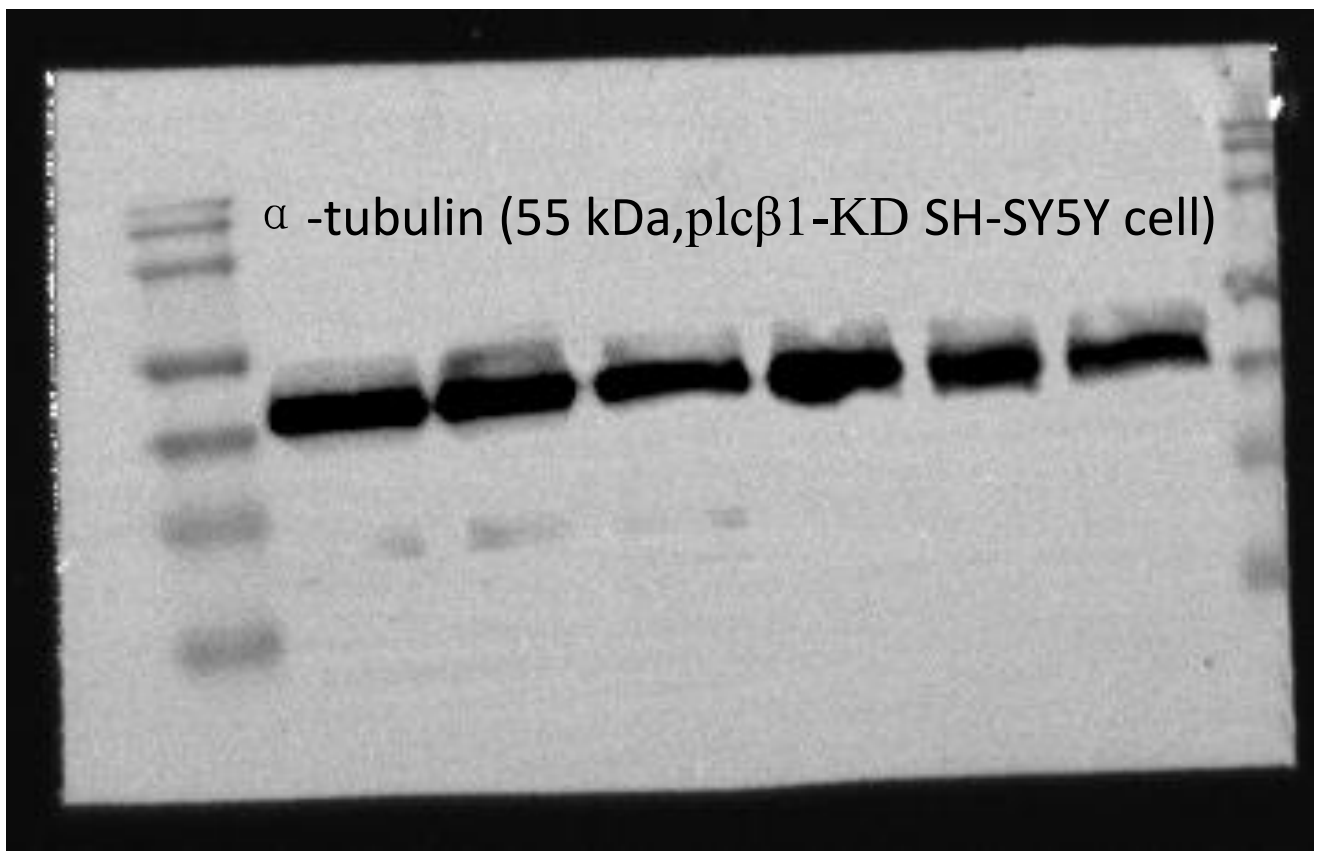
α -tubulin (55 kDa,plcβ1-KD SH-SY5Y cell)

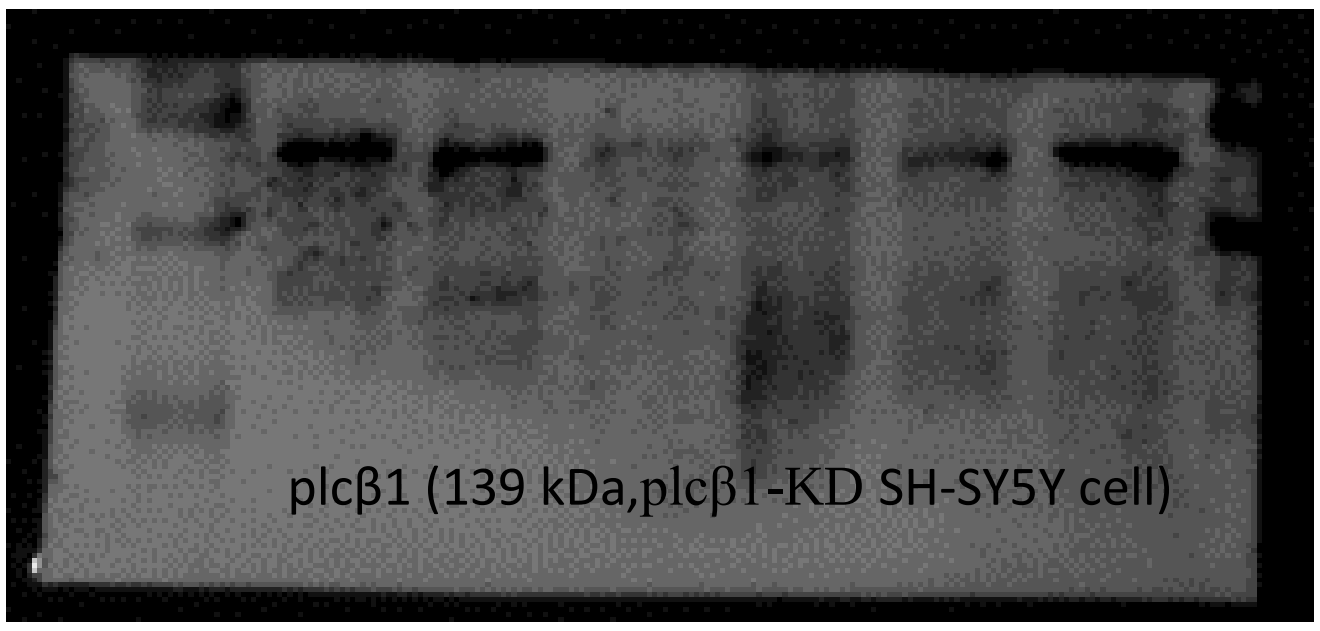
plcβ1 (139 kDa,plcβ1-KD SH-SY5Y cell)

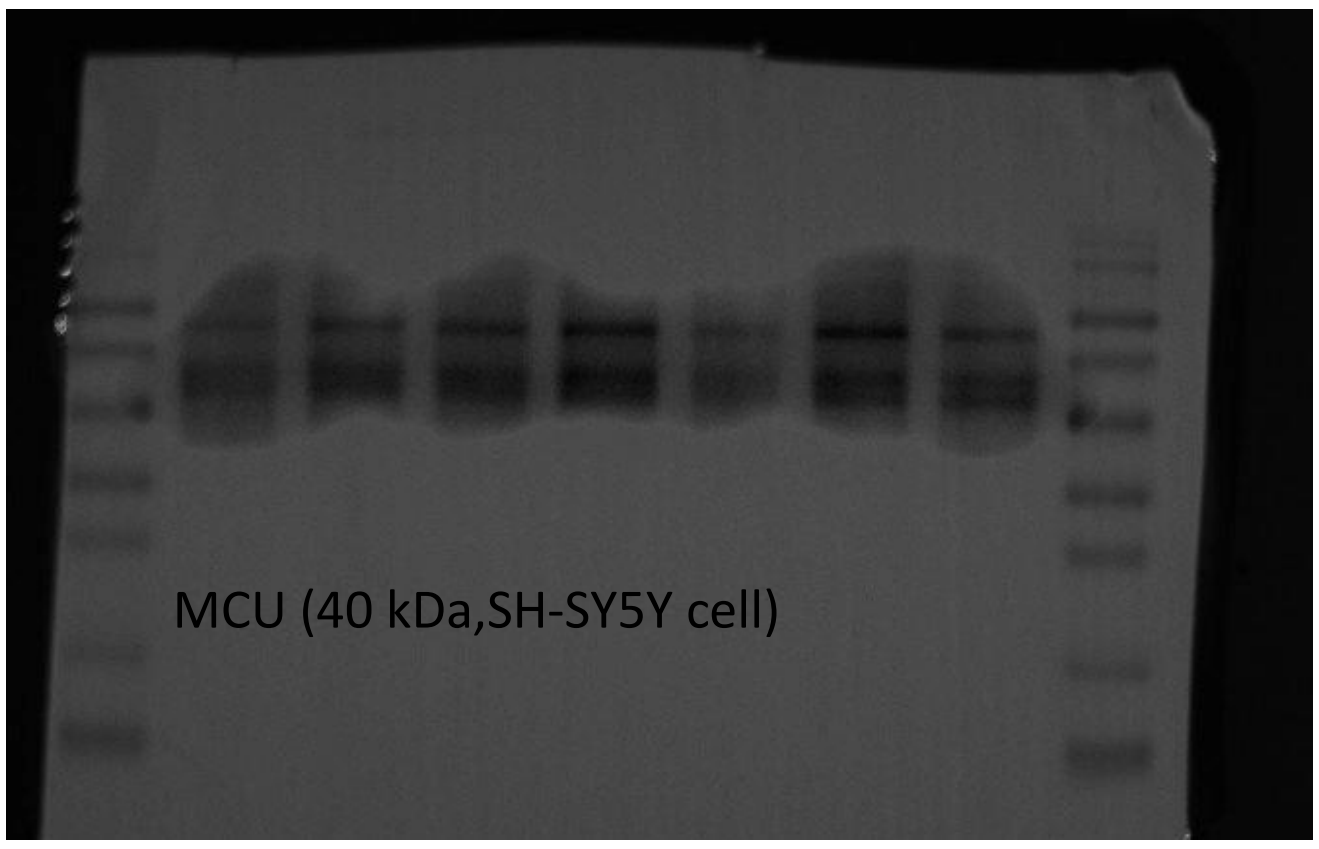
MCU (40 kDa,SH-SY5Y cell)
